# Associations of current and life-course smoking and indoor environmental exposures with lung function level in early adulthood

**DOI:** 10.1097/EE9.0000000000000523

**Published:** 2026-08-18

**Authors:** Diana M. Hendrickx, Gerard H. Koppelman, Judith M. Vonk, Jolanda M.A. Boer, Hans Jacob L. Koefoed, Marieke Oldenwening, Roel C.H. Vermeulen, Ulrike Gehring

**Affiliations:** a Institute for Risk Assessment Sciences, Utrecht University, Utrecht, The Netherlands; b Department of Paediatric Pulmonology and Paediatric Allergology, University Medical Center Groningen, Beatrix Children’s Hospital, University of Groningen, Groningen, The Netherlands; c University Medical Center Groningen, Groningen Research Institute of Asthma and COPD, University of Groningen, Groningen, The Netherlands; d Department of Epidemiology, University Medical Center Groningen, University of Groningen, Groningen, The Netherlands; e Centre for Prevention, Lifestyle and Health, National Institute for Public Health and the Environment, Bilthoven, The Netherlands; f Julius Centre for Health Sciences and Primary Care, University Medical Centre Utrecht, Utrecht, The Netherlands

**Keywords:** smoking, indoor environmental exposures, lung function, spirometry, longitudinal data

## Abstract

**Background:**

Lung function, expressed as forced expiratory volume in 1 second (FEV_1_), reaches its maximum at 20–30 years of age. A lower maximal lung function has been associated with a higher risk of chronic obstructive pulmonary disease in later adulthood, but the impact of smoking and indoor environmental exposures on this maximum remains unclear.

**Methods:**

Using data from 858 participants of the Dutch PIAMA birth cohort (mean age: 25.7 years), we investigated associations between smoking, second-hand smoke (SHS), dampness or mold, furry pets, and gas cooking exposure with maximal lung function. Exposure data were collected via questionnaires from pregnancy through early adulthood, and life-course exposure trajectories were derived using latent class growth modeling. We assessed associations with FEV_1_ and forced vital capacity (FVC) using linear regression, adjusting for potential confounders.

**Results:**

Pre- and post-bronchodilator spirometry was available for 846 and 806 participants, respectively. We found no associations between exposure to dampness or mold, furry pets, or gas cooking and lung function. Current and life-course active smoking was associated with higher post-bronchodilator FEV_1_, and pre- and post-bronchodilator FVC [regression coefficient β for pre-bronchodilator FVC (ml) (95% confidence interval): current (yes vs. no) 175 (68, 281), life-course (regular vs. occasional or none) 168 (55, 281)]. Current SHS was positively associated with post-bronchodilator FVC.

**Conclusion:**

No associations were found between indoor environmental exposures and maximal lung function in early adulthood. The observed positive associations with active smoking likely reflect bias arising from a selective uptake of smoking by individuals with higher pre-existing lung function, rather than a true physiological effect.

**What this study adds**

The article describes the relationships of life-course exposure trajectories and current exposure to SHS, dampness or mold, furry pets, and gas cooking, and the maximum lung function level in early adulthood in our prospective Dutch PIAMA (Prevention and Incidence of Asthma and Mite Allergy) birth cohort study. None of the exposures studied was associated with a lower lung function level in early adulthood. The availability and inclusion of exposure data for the entire period from birth until early adulthood is a major strength of our study compared with previous studies that generally focused on relatively short exposure periods.

## Background

Chronic obstructive pulmonary disease (COPD) is a leading cause of global mortality,^1^ responsible for 3.5 million deaths in 2021.^2^ COPD risk is higher in males than in females,^1^ reflecting differences in both biological (e.g., airway growth^3^) and environmental factors (e.g., historically higher smoking rates in males^4^). Key risk factors include tobacco smoke, indoor and outdoor exposures^5^ [e.g., second-hand smoke (SHS), gas cooking,^6^ and allergens from furry pets^6^].

COPD is typically diagnosed after age 40,^7^ but about half of COPD patients have been found to have a low maximally attained lung function in early adulthood.^8^ Lung function increases throughout childhood and adolescence and reaches its maximum in early adulthood (age 20–30 years).^9,10^ Because a low maximal lung function in early adulthood is a strong determinant of COPD development later in life, identifying determinants of low maximal lung function is crucial for prevention.

Longitudinal studies examining the impact of indoor air pollution across the entire life course on early adulthood lung function are scarce. The Tasmanian Longitudinal Health Study linked maternal smoking during childhood to trajectories with low lung function in early adulthood.^11^ However, most research has focused on limited exposure periods, and not the full span from birth to adulthood.

Building on research by Milanzi et al^12^ who found that childhood SHS exposure was associated with lower lung function in adolescence and that early-life pet exposure may reduce lung function growth, this study investigates associations between current and life-course exposure to tobacco smoke, SHS, dampness or mold, furry pets, and gas cooking with maximal lung function [forced expiratory volume in 1 second (FEV_1_) and forced vital capacity (FVC)], and clinically defined low lung function in early adulthood. We hypothesize that both current and life-course exposures are associated with lower maximum lung function.

## Methods

### Study design and population

We used data from 858 participants of the Dutch Prevention and Incidence of Asthma and Mite Allergy (PIAMA) cohort, who participated in the most recent medical examination at a mean age of 25 years between October 2021 and February 2024, to determine trajectories of life-course exposure. The PIAMA cohort has been described elsewhere in detail.^13^ In brief, the baseline study population consisted of 3963 children born in 1996 and 1997, recruited prenatally in the northern, central, and western regions of the Netherlands. Data on demographics, indoor exposures, lifestyle, and health characteristics were collected by parent- and participant-completed questionnaires. Parental questionnaires were administered prenatally (during pregnancy) and postnatally (child’s age of 3 months, annually from 1 up to 8 years, and at 11, 14, and 17 years). Participants themselves completed questionnaires every 3 years from age 11 up to 23 years. The subset of questions used to assess smoking and indoor exposures in this study is provided in Supplementary Appendix A, https://links.lww.com/EE/A462. Lung function was measured at a mean age of 25 years. The current study population consists of participants with lung function measurements at a mean age of 25 years, and data on active smoking and SHS, dampness or mold, pets, and/or gas cooking exposure from at least one survey.

### Ethics approval and consent to participate

This research was conducted in accordance with the Declaration of Helsinki and was approved by the institutional review board (METC Utrecht, dossier number NL74439.041.20, METC protocol number 20-490/M). Written informed consent was obtained from the parents or legal guardians of all participants and from the participants themselves.

### Lung function measurements

Maximally attained FEV_1_ and FVC were measured by spirometry at a mean age of 25 years before and 15 minutes after the intake of a bronchodilator (400 µg of salbutamol via a metered dose inhaler with spacer), using an EasyOne Pro LAB spirometer (NDD Medical Technologies, Switzerland, https://nddmed.com/products/complete-pulmonary-function-machine/easyone-pro-lab/). Spirometry was performed according to the American Thoracic Society/European Respiratory Society recommendations^14^ mainly in Utrecht (N = 829) and exceptionally in Groningen (N = 29). All flow volume curves were reviewed by a highly experienced lung function technician. At least three acceptable maneuvers were required for each participant, and the maximum FEV_1_ and FVC were derived from these if the differences between the largest and next largest values for FEV_1_ and FVC were ≤200 ml or 5%. The main outcomes were FEV_1_ and FVC. For some of the 858 subjects, lung function measurements were also available at ages 8 (n = 348), 12 (n = 543), and 16 (n = 399) years as described previously.^15,16^ In brief, lung function was measured using Jaeger Masterscreen pneumotachographs (Viasys Healthcare, Conshohocken, PA) at the age of 8 years and EasyOne spirometers (Medical Technologies Inc., Zurich, Switzerland) at age 12, and both devices at age 16.

Clinically low lung function was defined as having an FEV_1_ below the lower limit of normal (LLN), that is, the 5th percentile of test results from a reference population [Global Lung Function Initiative (GLI) reference equations].^17^

### Exposure assessment

#### Active smoking

Information on active smoking has been collected from age 11 until 23 years, and active smoking was defined as smoking at least once a week (yes/no).

#### Second-hand smoke exposure

SHS exposure was assessed from pregnancy until age 23 years. SHS during pregnancy was defined as maternal smoking during at least the first 4 weeks of pregnancy. From 3 months until age 23, SHS exposure was defined as anyone smoking at least once a week in the participant’s home (yes/no).

#### Exposure to dampness or mold

Information on exposure to dampness or mold was collected from age 3 months until age 23. It was restricted to exposure in the rooms where the participants were expected to spend most of their time: the living room and the participant’s bedroom. We used the question “Have you seen any moisture stains or mould on the ceiling or walls in the last 12 months?” and dichotomized dampness or mold exposure as yes (answer is “yes” for the living room and/or the participant’s bedroom) and no (answer is “no” for both the living room and the participants’ bedroom).

#### Exposure to furry pets

Exposure to furry pets was available from pregnancy to age 23 years, using the question “Do you keep a dog/cat/rodent indoors?” (yes/no). Cat and dog exposure were also assessed separately as associations with lung function may differ for cats and dogs.^18^

#### Gas cooking

Use of gas for cooking (yes/no) was assessed from age 3 months to age 23. If information on gas cooking was missing in a questionnaire, and this participant had not changed address between this and the previous or subsequent questionnaire, the value from the previous or subsequent questionnaire was carried forward/backward, respectively.

### Potential covariates

Figure S1, https://links.lww.com/EE/A462, shows a directed acyclic graph illustrating potential covariates identified by a review of relevant literature, and assumptions about their relationships with active smoking, indoor exposures and lung function. Sex (at birth), age, height, and parental country of birth [The Netherlands (yes/no)] were included as predictors of lung function in the minimally adjusted models.^14^ The following other factors were additionally considered a priori as potential confounders in the additionally adjusted models: a self-reported respiratory infection in the last three weeks before the spirometric measurement as this may affect lung function,^12^ parental allergy (defined as positive if one of the parents ever had asthma, was allergic to house dust, house dust mite or pets, or had hay fever) as this may be associated with allergy of the participant and (avoidance) of exposures,^19,20^ highest education of the parents and the participant as indicators of socioeconomic status.^21^ Education of the parents was defined as the highest education reported by either parent. Education of the participant was defined as the highest completed education (for nonstudents) or current education (for students). Education was categorized into “low,” “middle,” and “high” according to the Statistics Netherlands (Centraal Bureau voor de Statistiek) classification.^22^

### Missing value imputation

Missing values for the parental country of birth were imputed with the most common value. In the case that the education of one parent was missing, the education of the other parent was used. In the case that education of both parents was missing, the missing value was imputed by the most common value.

For all ages, missing values on active smoking and exposure to SHS, dampness or mold, pets, and gas cooking in one or more of the surveys were imputed in R 4.4.0^23^ with the mice package^24^ (version 3.16.0), using the default settings. Five imputed data sets were created, and the results of the statistical analysis were pooled using Rubin’s rules.^25^

### Statistical analysis

Study population characteristics were calculated on the nonimputed data and are provided as percentages of the total number of observations for categorical variables and mean ± standard deviation (SD) for continuous variables.

Current exposure was defined based on the survey conducted at age 23 years. We analyzed single-exposure models, as well as multi-exposure models including all five exposures (active smoking, SHS, dampness or mold, pets, and gas cooking). Potential multicollinearity problems in multi-exposure models were assessed with pairwise correlations among indoor exposures and variance inflation factors.

We characterized life-course exposure to active smoking, SHS, dampness or mold, pets, and gas cooking by longitudinal exposure trajectories using latent class growth modeling (LCGM).^26^ LCGM was implemented in R 4.3.2^23^ using the lcmm package^27^ (version 2.0.2). We considered linear, quadratic, and cubic models with 1–6 classes (trajectories). To avoid convergence to a local optimum when estimating the parameters of the models with more than one class, we randomly generated 1500 sets of initial parameter values from the asymptotic distribution of the parameter estimates of the one-class model of the same order. The choice of the order and number of classes in the final model was based on the Bayesian Information Criterion and having a class membership >4% for all groups. LCGM generates posterior class probabilities (between 0 and 1) for each trajectory, which were included as continuous independent variables in the statistical analysis except for one trajectory that was used as a reference. All 858 subjects who participated in the medical exam at a mean age of 25 were included in the LCGM procedure. Table S1, https://links.lww.com/EE/A462, presents the percentage of surveys with missing values for the different exposures. Trajectories were based on imputed data.

Minimally and additionally adjusted linear regression analyses were performed to assess associations of current exposure and life-course exposure trajectories with FEV_1_ and FVC at a mean age of 25 years. We also investigated associations of current exposure and life-course exposure trajectories with clinically defined low lung function (yes/no) using logistic regression models. As the calculation of the LLN already takes into account sex, age, height, and parental country of birth,^17^ we did not adjust for these covariates in the regression model and only further adjusted the models for all other confounders mentioned before.

Statistical analyses were performed in R version 4.4.0^23^ at a 0.05 level of significance.

### Effect modification by sex

As the development of COPD has been found to differ by sex,^28^ we tested for exposure–sex interactions by including interaction terms between the exposures and sex in the models and performed stratified analysis by sex for all exposures.

### Sensitivity analysis

To investigate whether avoidance of smoking and indoor exposures by (parents of) subjects with asthma distorted associations of exposures with FEV_1_ and FVC at mean age 25 years, we repeated the pre-bronchodilator analyses excluding participants with asthma at mean age 25 years. We also repeated the analysis excluding participants with asthma during the first 4 years of life, and excluding participants with parental asthma. To assess whether participants with a lower lung function are less likely to start smoking, we compared lung functions at mean age 25 years between current and never-smokers. We also compared lung function measurements at ages 8, 12, and 16 years available for our study sample between participants who did and did not become smokers by age 23.

## Results

### Population characteristics

Of the 858 participants, 846 and 806, respectively, had pre-and post-bronchodilator measurements that passed quality control. A flowchart with the number of participants in each analysis is presented in Figure S2, https://links.lww.com/EE/A462.

Study population characteristics and summary statistics for the lung function measurements are presented in Table 1. Sixty-four percent had at least one highly educated parent, 31% had an atopic mother, and 36% had an atopic father. As only 10 participants reported low education (Table 1), we combined “low” and “middle” education of the participants into a single group. The current study population showed a lower percentage of boys, and higher percentages of participants with atopic fathers and highly educated parents than the baseline PIAMA cohort (Table S2, https://links.lww.com/EE/A462).

**Table 1.** Study population characteristics (before missing data imputation)

Covariates	Study population trajectories (N = 858)^a^	Study population pre-bronchodilator (N = 846)^a^	Study population post-bronchodilator (N = 806)^a^
FEV_1_ (ml), mean ± SD		4,030 ± 784^b^	4,177 ± 809^c^
FVC (ml), mean ± SD		4,952 ± 1,041^b^	4,940 ± 1,042^c^
FEV_1_ % predicted, mean ± SD		95.9 ± 10.3	99.4 ± 9.9
FVC % predicted, mean ± SD		98.9 ± 10.1	98.8 ± 10.2
% Improvement in FEV_1_ after intake bronchodilator, mean ± SD^d^	3.9 ± 3.9 (N = 800)		
% Improvement in FVC after intake bronchodilator, mean ± SD^d^	−0.2 ± 2.3 (N = 800)		
Prevalence of FEV_1_ <LLN, n/N (%)		60/846 (7.1%)	20/806 (2.5%)
Prevalence of FVC <LLN, n/N (%)		24/846 (2.8%)	27/806 (3.3%)
Prevalence of asthma, n/N (%)	74/858 (8.6%)	74/846 (8.7%)	72/806 (8.9%)
Male, n/N (%)	343/858 (40.0%)	338/846 (40.0%)	319/806 (39.6%)
Parental country of birth (The Netherlands), n/N (%)	782/844 (92.7%)	770/832 (92.5%)	734/793 (92.6%)
Respiratory infections^e^, n/N (%)	241/858 (28.1%)	239/846 (28.3%)	229/806 (28.4%)
Parental allergy, n/N (%)			
Allergy mother	269/858 (31.4%)	267/846 (31.6%)	258/806 (32.0%)
Allergy father	304/856 (35.5%)	300/844 (35.5%)	284/804 (35.3%)
Parental education, n/N (%)			
Low	58/848 (6.8%)	56/836 (6.7%)	55/797 (6.9%)
Intermediate	247/848 (29.1%)	244/836 (29.2%)	231/797 (29.0%)
High	543/848 (64.0%)	536/836 (64.1%)	511/797 (64.1%)
Education participant, n/N (%)			
Low	10/849 (1.2%)	10/837 (1.2%)	9/797 (1.1%)
Intermediate	138/849 (16.3%)	134/837 (16.0%)	129/797 (16.2%)
High	701/849 (82.6%)	693/837 (82.8%)	659/797 (82.7%)
Age (years), mean ± SD	25.7 ± 0.6	25.7 ± 0.6	25.7 ± 0.6
Height (cm), mean ± SD	176.9 ± 9.0	176.9 ± 9.0	176.9 ± 9.0

a For some covariates N is lower than the study population size because of missing data.

b Pre-bronchodilator FEV_1_ and FVC,

c Post-bronchodilator FEV1 and FVC.

d For the population with a reproducible pre- and post-bronchodilator measurement (N = 800).

e Respiratory infection in the last 3 weeks before; spirometric measurements.

Prevalence of exposure at different ages is presented in Table S3, https://links.lww.com/EE/A462. During the first 3 months of life, 22.3% of the participants were exposed to SHS, 9.6% to dampness or mold, 42.4% to furry pets, and 83.5% to gas cooking (Table S3, https://links.lww.com/EE/A462). At the age of 23 years, 4.5% of the participants were exposed to SHS, 13.9% to dampness or mold, 33.7% to furry pets, and 64.8% to gas cooking (Table S3, https://links.lww.com/EE/A462). The mean ± SD values of the pre-bronchodilator FEV_1_ and FVC at a mean age of 25 years were 4,030 ± 784 and 4,952 ± 1041 ml, respectively.

Distributions of study characteristics by current exposure status are shown in Tables S4–S8, https://links.lww.com/EE/A462.

### Association of current exposures with the maximum FEV_1_ and FVC

Minimally adjusted (Table S9, https://links.lww.com/EE/A462) and additionally adjusted associations (Table 2) of current smoking and indoor exposures with pre-bronchodilator and post-bronchodilator FEV_1_ and FVC were similar. The results of the single-exposure models showed positive associations between active smoking and pre- and post-bronchodilator FVC and post-bronchodilator FEV_1_. For example, for the additionally adjusted model, the estimate for active smoking (yes vs. no) for the pre-bronchodilator FVC was 175 ml [95% confidence interval (CI): 68, 281 ml] (Table 2). SHS exposure was positively associated with post-bronchodilator FVC [e.g., for the additionally adjusted model, the estimate for SHS (yes vs. no) for the post-bronchodilator FVC was 253 ml (95% CI: 79, 426 ml)] (Table 2). No significant associations of exposure to dampness or mold, pets, and gas cooking with FEV_1_ and FVC were observed (Tables 2 and S9, https://links.lww.com/EE/A462).

**Table 2.** Additionally adjusted^a^ associations of current active smoking and indoor exposures with lung function

Exposure (yes vs. no)	FEV_1_: difference (ml) (95% CI)		FVC: difference (mL) (95% CI)	
	Pre-bronchodilator (n = 846)	Post-bronchodilator (n = 806)	Pre-bronchodilator (n = 846)	Post-bronchodilator (n = 806)
Active smoking	56 (−37, 150)	**112 (21, 202**)	**175 (68, 281**)	**186 (77, 296**)
SHS	33 (−114, 181)	128 (−15, 272)	155 (−15, 325)	**253 (79, 426**)
Dampness or mold	13 (−75, 100)	18 (−67, 103)	−20 (−120, 80)	−1 (−104, 101)
Furry pets (total)	−39 (−105, 28)	−26 (−91, 39)	−14 (−90, 61)	−5 (−83, 73)
Cats	−57 (−131, 17)	−45 (−118, 27)	−9 (−96, 78)	1 (−88, 90)
Dogs	−9 (−99, 82)	5 (−83, 93)	12 (−90, 114)	8 (−98, 114)
Gas cooking	−17 (−80, 47)	−40 (−102, 22)	−45 (−118, 27)	−62 (−137, 13)

Associations from single-exposure models, expressed as estimated difference (ml) between exposed and nonexposed participants. Bold = *P* value <0.05.

a Adjusted for sex, height, age, parental country of birth, parental allergy, cold, or respiratory infection in the past 3 weeks, highest education of the parents, and highest education of the participant.

The results of the multi-exposure models were similar to those of the single-exposure models, except for the association between gas cooking and post-bronchodilator FVC, which was negative in the multi-exposure model (Table S10, https://links.lww.com/EE/A462). Pairwise correlations among indoor exposures were modest (active smoking-SHS φ = 0.27; active smoking-gas cooking φ = 0.10; all others <0.1, Table S11, https://links.lww.com/EE/A462), and variance inflation factors were <2 (Table S12, https://links.lww.com/EE/A462), indicating that collinearity was not a concern. Comparison of the multi-exposure model with the two-exposure model for gas cooking and active smoking shows that the shift in association from nonsignificant to negative already occurs in the two-exposure model (Table S13, https://links.lww.com/EE/A462), suggesting that the estimate of this association may have been influenced by the moderate correlation between smoking and gas cooking.

We did not study associations with rodents separately as the number of participants with rodents was insufficient (only 42 of the 858 subjects who participated in the most recent medical exam).

### Associations of current exposures with clinically defined low lung function

Of the 846 participants with successful pre-bronchodilator lung function measurements at mean age 25 years, 60 (7.1%) were identified as having a clinically defined low lung function (pre-bronchodilator FEV_1_ below LLN). This is in line with the general population, for which it is reported that 4%–12% of young adults never achieve a normal maximum FEV_1_.^29^ None of the exposures was associated with a clinically low lung function; minimally adjusted (Table S14, https://links.lww.com/EE/A462) and additionally adjusted associations (Table 3) of current smoking and indoor exposures with clinically low lung function were similar.

**Table 3.** Associations of current active smoking and indoor exposures with clinically low lung function

Exposure (yes vs no)	Odds ratio (95% CI) clinically low lung function	
	Minimally adjusted^a^ (n = 846)	Additionally adjusted^b^ (n = 846)
Active smoking	1.54 (0.75, 3.14)	1.51 (0.73, 3.11)
SHS	2.14 (0.80, 5.72)	2.22 (0.82, 6.00)
Dampness or mold	1.12 (0.54, 2.35)	1.12 (0.53, 2.35)
Furry pets (total)	1.00 (0.56, 1.78)	1.07 (0.59, 1.96)
Cats	1.30 (0.70, 2.39)	1.34 (0.71, 2.50)
Dogs	0.53 (0.19, 1.43)	0.57 (0.21, 1.58)
Gas cooking	1.46 (0.80, 2.64)	1.46 (0.80, 2.65)

Associations from single-exposure models, expressed as estimated odds ratio between exposed and nonexposed participants. Clinically low lung function was defined as having an pre-bronchodilator FEV_1_ below the lower limit of normal (LLN).

a Adjustments for sex, height, age and ethnicity included in GLI equations.

b Adjusted for parental allergy, cold or respiratory infection in the past 3 weeks, highest education of the parents, and highest education of the participant.

Of the 846 participants with successful pre-bronchodilator lung function measurements, only 24 (2.8%) had a pre-bronchodilator FVC below LLN. Of the 806 participants with successful post-bronchodilator lung function measurements, only 20 (2.5%) had a post-bronchodilator FEV_1_ below LLN. Logistic regression analyses were therefore restricted to clinically low pre-bronchodilator FEV_1_, for which the prevalence was sufficient (7.1%) to allow model convergence.

### Exposure trajectories

**Figure 1.**
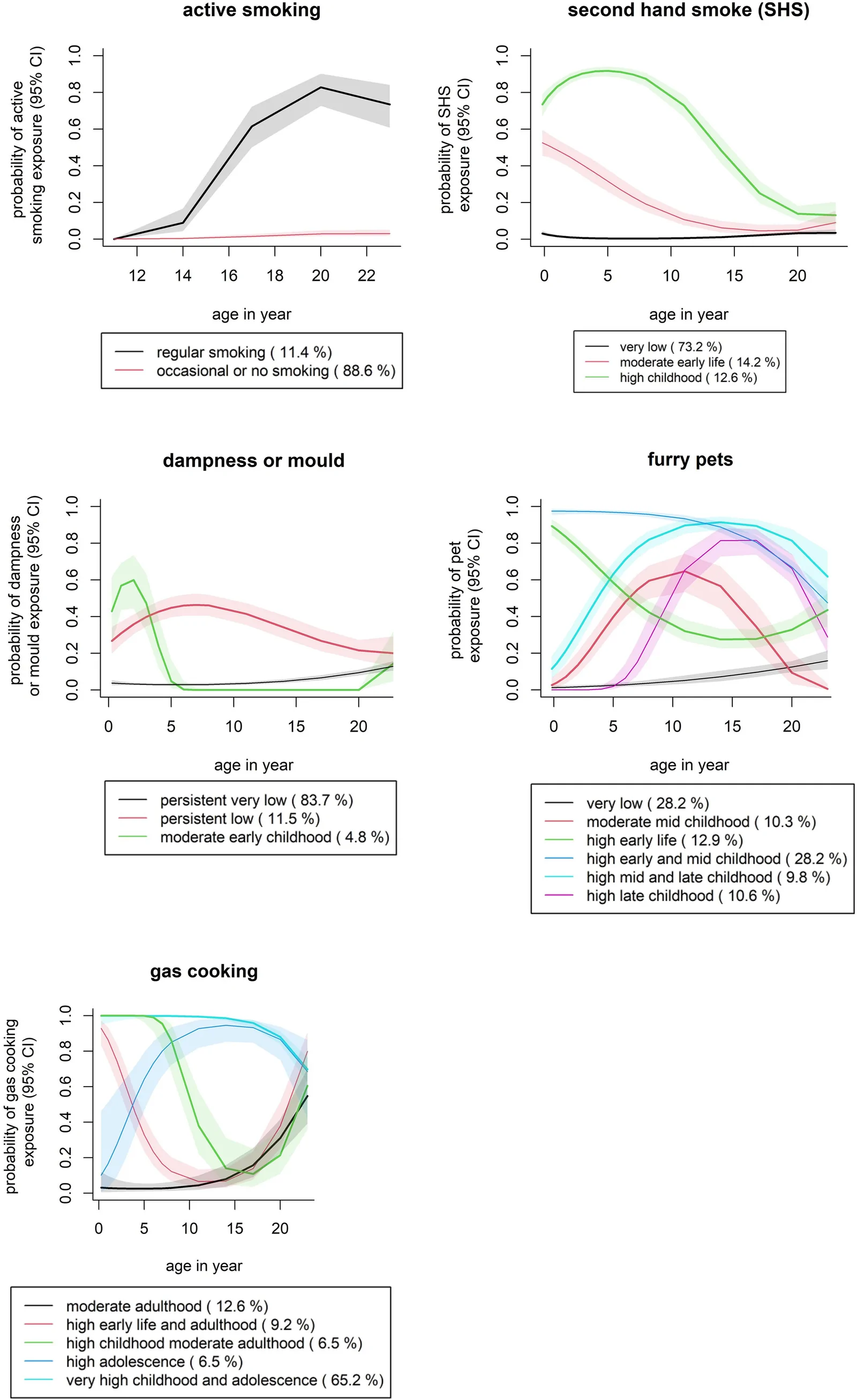
Longitudinal trajectories of active smoking and indoor exposures with trajectory frequencies.

We identified two-exposure trajectories for active smoking, three trajectories each for SHS and dampness or mold, six for furry pets exposure and five for gas cooking (Figure 1). For all exposures, a trajectory characterized by very low or low probability of exposure throughout childhood was observed. In addition, for all exposures, except active smoking, which was assessed from age 11 onward, we identified a trajectory with moderate or high probability of exposure in early life. When cat and dog exposure were analyzed separately, five longitudinal trajectories were identified for cat exposure and four for dog exposure (Figure S3, https://links.lww.com/EE/A462). A trajectory representing high early-life exposure was observed only for cats; the remaining trajectories were similar between cats and dogs. Distributions of participant characteristics across exposure trajectories are presented in Tables S15–S19, https://links.lww.com/EE/A462.

### Associations of exposure trajectories with the maximum FEV_1_ and FVC

Minimally adjusted (Table S20, https://links.lww.com/EE/A462) and additionally adjusted associations (Table 4) of exposure trajectories with pre- and post-bronchodilator FEV_1_ and FVC were similar. We observed higher pre- and post-bronchodilator FVC and a higher post-bronchodilator FEV_1_ in the regular smoking trajectory compared to the occasional or nonsmoking trajectory. For example, for the additionally adjusted model, the estimate for regular smoking compared with occasional or no smoking for the pre-bronchodilator FVC was 168 ml (95% CI: 55, 281 ml) (Table 4). No associations of trajectories of SHS, dampness or mold, pets, cat, dog, and gas cooking exposures with pre- and post-bronchodilator FEV_1_ and FVC were observed (Table 4).

**Table 4.** Additionally adjusted associations of longitudinal trajectories of active smoking and indoor exposures with lung function

Exposure		FEV_1_: difference (ml) (95% CI)		FVC: difference (ml) (95% CI)	
		Pre-bronchodilator (n = 846)	Post-bronchodilator (n = 806)	Pre-bronchodilator (n = 846)	Post-bronchodilator (n = 806)
Active smoking	Regular smoking vs. occasional or no smoking	60 (−38, 158)	**101 (5, 196**)	**168 (55, 281**)	**173 (59, 288**)
SHS	Moderate early life vs. very low	38 (−58, 133)	36 (−57, 129)	55 (−55, 165)	50 (−63, 162)
	High childhood vs. very low	44 (−53, 141)	49 (−47, 146)	33 (−79, 145)	32 (−85, 148)
Dampness or mold	Persistent low vs. persistent very low	−4 (−107, 99)	−14 (−113, 86)	3 (−113, 118)	1 (−118, 119)
	Moderate early childhood vs. persistent very low	39 (−135, 213)	32 (−141, 204)	162 (−38, 362)	115 (−92, 321)
Furry pets (total)	Moderate mid-childhood vs. very low	19 (−115, 153)	23 (−109, 154)	0 (−155, 155)	38 (−122, 199)
	High early life vs. very low	0 (−109, 110)	−7 (−114, 100)	37 (−87, 162)	19 (−109, 147)
	High early and mid−childhood vs. very low	−5 (−89, 78)	−20 (−103, 62)	−9 (−106, 88)	−25 (−125, 75)
	High mid- and late childhood vs. very low	−75 (−195, 45)	−79 (−199, 40)	−93 (−232, 47)	−72 (−218, 74)
	High late childhood vs. very low	−17 (−142, 107)	8 (−114, 131)	21 (−125, 168)	35 (−115, 185)
Cats	High early life vs. persistent very low	9 (−101, 120)	−15 (−123, 93)	5 (−123, 132)	−15 (−146, 116)
	High early and mid-childhood vs. persistent very low	20 (−59, 100)	12 (−66, 90)	−18 (−110, 74)	−22 (−117, 72)
	High mid- and late childhood vs. persistent very low	−19 (−152, 113)	−21 (−152, 110)	−69 (−221, 82)	−61 (−218, 96)
	High late childhood vs. Persistent very low	21 (−99, 141)	1 (−115, 117)	−4 (−139, 138)	12 (−130, 153)
Dogs	Moderate mid-childhood vs. persistent very low	−48 (−181, 85)	−25 (−156, 106)	11 (−145, 167)	26 (−133, 185)
	High early childhood vs. persistent very low	−57 (−150, 36)	−61 (−152, 31)	−18 (−127, 90)	−37 (−149, 75)
	High adolescence vs. persistent very low	−20 (−115, 75)	−16 (−110, 79)	−33 (−145, 79)	−28 (−144, 89)
Gas cooking	High early life and adulthood vs. moderate adulthood	−90 (−228, 48)	−90 (−225, 45)	−58 (−219, 103)	−69 (−249, 111)
	High childhood moderate adulthood vs. moderate adulthood	−54 (−203, 96)	4 (−141, 148)	0 (−171, 170)	35 (−140, 209)
	High adolescence vs. moderate adulthood	−110 (−268, 48)	−85 (−246, 76)	−64 (−252, 125)	−39 (−239, 162)
	Very high childhood and adolescence vs. moderate adulthood	−47 (−140, 45)	−35 (−128, 58)	−5 (−114, 104)	−3 (−119, 118)

Models were adjusted for sex, height, age, parental country of birth, parental allergy, cold, or respiratory infection in the past 3 weeks, highest education of the parents, and highest education of the participant. Associations expressed as estimated difference (ml) between trajectories. Bold = *P* value <0.05.

### Associations of exposure trajectories with clinically low lung function

No associations between exposure trajectories of active smoking and indoor exposures and clinically low lung function were found (Table S21, https://links.lww.com/EE/A462). Minimally adjusted and additionally adjusted associations of exposure trajectories with clinically low lung function were similar (Table S21, https://links.lww.com/EE/A462).

### Effect modification by sex

No interactions of sex with current active smoking, SHS, furry pets, and gas cooking exposure were found (Table S22, https://links.lww.com/EE/A462). For current exposure to dampness or mold, the associations with FEV_1_ and FVC were nonsignificant, but their directionality differed by sex, showing positive associations among males and negative associations among females (Table S22, https://links.lww.com/EE/A462).

Similarly, associations of longitudinal trajectories of active smoking and indoor exposures with pre-bronchodilator FEV_1_ and FVC at mean age 25 years did not differ by sex (Table S23, https://links.lww.com/EE/A462).

### Sensitivity analyses

Sensitivity analysis excluding participants with asthma at age 23 (n = 74) showed that the associations of current and life-course active smoking and indoor exposures with pre-bronchodilator FEV_1_ and FVC did not change (Tables S24 and S25, https://links.lww.com/EE/A462). Similarly, excluding participants with asthma during the first 4 years of life (n = 140) did not change associations of current and life-course active smoking and SHS indoor exposures with FEV_1_ and FVC (Tables S26 and S27, https://links.lww.com/EE/A462). Associations also remained similar after excluding participants with parental asthma (n = 139) (Tables S28 and S29, https://links.lww.com/EE/A462). Former smokers had similar and never-smokers had lower lung functions than current smokers (Table S30, https://links.lww.com/EE/A462). Additional analyses of lung function data at ages 8, 12, and 16 years within our study sample showed that participants who became smokers by age 23 already had higher lung function at ages 12 and 16 years, but not at age 8 years (Table S31, https://links.lww.com/EE/A462).

## Discussion

Exposure to dampness or mold, furry pets, and gas cooking was not associated with FEV_1_, FVC, or low lung function in early adulthood. Current and life-course smoking were associated with higher pre- and post-bronchodilator FVC and post-bronchodilator FEV_1_, while current, but not life-course, SHS exposure was associated with higher post-bronchodilator FVC.

This research extends earlier research within the PIAMA cohort at mean ages 12.6 and 16.3 by Milanzi et al,^12^ who reported that SHS and dampness or mold were associated with lower lung function and pets with higher lung function. The associations in adolescence were not observed in the present analysis in early adulthood. This discrepancy may reflect lung function catch‑up associated with the attainment of maximal lung function in early adulthood,^30^ reduced exposure variability at later ages, or selective retention of healthier participants. Sensitivity analyses excluding participants with asthma or parental asthma did not significantly change the results, suggesting that selective retention of healthy individuals is unlikely to explain the disappearance of associations. Our analysis suggests that persistent or current smoking is linked to higher FVC, independent of sex and asthma status, indicating that reverse causation from smoking avoidance by asthmatics is unlikely to explain these positive associations. However, the “healthy smoker” effect—selective continuation of smoking among less symptomatic individuals^31^—remains possible. Unfortunately, information on reasons for quitting or never initiating smoking was not available.

Former smokers had similar, and never-smokers had lower lung function than current smokers (Table S27, https://links.lww.com/EE/A462), strengthening the hypothesis that people with a lower lung function may be less likely to start smoking. Similar findings have been reported previously.^32–34^ Explanations include higher baseline lung function among smokers,^34^ lung expansion due to inhalation,^33^ or misreporting smoking status, though the lack of a corresponding FEV_1_ increase argues against the latter. Additional analyses of lung function data at ages 8, 12, and 16 years within our study sample showed that participants who became smokers by age 23 already had higher lung function at ages 12 and 16 years, but not at age 8 years, supporting the interpretation that the positive association between lung function at age 25 and smoking may reflect at least in part bias arising from selective uptake of smoking by individuals with higher pre‑existing lung function rather than a physiological effect. Positive associations between smoking and FVC but not FEV_1_ may reflect differences in physiological aspects captured by FEV_1_ and FVC: FEV_1_ primarily reflects airflow limitation, whereas FVC represents total lung volume.^35^

In contrast to our findings, previous studies reported similar or lower FEV_1_ and FVC in SHS-exposed young adults.^36–38^ One study found a higher FVC in adolescents with smoking parents, which disappeared after adjusting for other SHS sources.^39^ Exposure misclassification is a potential concern, but prior validation in PIAMA using home nicotine measurements supported the reliability of self-reported data.^40,41^

Dampness or mold exposure was not associated with lung function, although associations tend to be positive in men and negative in women. Previous adult studies (aged ≥18 years and 21–63 years, respectively) reported lower FEV_1_ and FVC in those exposed to dampness or mold^42,43^ and faster FEV_1_ decline among exposed women.^44^ Several physiological and behavioral mechanisms may contribute to sex‑specific patterns. Men have larger lungs and larger airways relative to lung size than women,^45,46^ while women show greater airway hyperresponsiveness,^47^ and therefore women may be more susceptible to dampness or mold than men. Behavioral differences, including more time spent indoors by women,^48^ may also influence exposure to dampness or mold. These mechanisms could contribute to sex‑specific patterns, although our findings should be interpreted cautiously given the lack of statistical significance.

For pets, our findings align with Dratva et al,^49^ who found no association between early-life pet exposure and adult (age 28–73) lung function. Our study extends this evidence specifically to early adulthood.

Consistent with previous adult studies (ages 20–44 and 20–74 years),^50,51^ negative associations of FEV_1_ with gas cooking, though nonsignificant, were stronger in women than in men.

Dai et al^52^ showed that adults exposed to gas cooking between ages 43 and 53 years showed a faster lung function decline during this period compared to adults using electric cooking. In contrast, a cohort of adults aged 34–35 years found no association between gas cooking in childhood and lung function,^53^ which corresponds with our results in early adulthood. In the same cohort, current gas cooking was negatively associated with FEV_1_, but not FVC, in men only.^53^ In our study, we found no evidence that current gas cooking or life-course gas cooking affected lung function, suggesting that potential adverse effects (if any) may emerge later in life.

Strengths of this study include the prospective cohort design, detailed exposure assessment from pregnancy or birth to early adulthood, and life-course trajectory analysis.

Potential limitations include self-reported exposure data, selective loss to follow-up, and potential underestimation of adult exposure, as only residential environments were considered. Compared with the original cohort, the analytic sample at mean age 25 contained fewer males and a higher proportion of highly educated families. Because highly educated families and participants are generally less exposed to SHS, dampness/mold, and pets,^54,55^ this selective retention may have reduced exposure variability, and consequently statistical power and our ability to detect associations. However, the overrepresentation of highly educated families was also observed in earlier analyses,^12^ where we observed associations between childhood SHS and mold exposure and adolescent lung function. This may affect the generalizability of our findings to the general Dutch population. To assess differences in exposure variability compared to previous analyses, we compared indoor exposure trajectories in our study with trajectories up to age 12 from our previous study in adolescents.^12^ For SHS and dampness or mold, our study identified three trajectories, while in our previous study, four trajectories were identified, which suggests less exposure variability in our sample. However, our previous study in adolescents reported a lower FEV_1_ for all SHS trajectories compared with the “very low” category. Thus, the fact that there are fewer trajectories does not explain why we see null or positive effect estimates in the current analysis. For pets, our study identified more trajectories than our previous research in adolescents (six vs. five), so there may actually be slightly more variation. However, that still does not explain why the observations of our previous study in adolescence were not observed in early adulthood. The above comparisons argue against selection and variability in exposure as explanations for the discrepancy between our current and our previous result, and that the third option, lung function catch‑up associated with the attainment of maximal lung function in early adulthood, remains a possible explanation. Moreover, LCGM inherently depends on the study population, meaning trajectories may differ in other cohorts. Finally, the 2-year gap between questionnaires and the medical exam (including spirometry) due to delays of medical exams by the COVID-19 pandemic left exposures at ages 23–25 years uncaptured. This 2‑year gap also introduces the possibility of exposure misclassification, as smoking behavior and housing conditions may change during early adulthood. Consequently, “current” exposure at age 23 may not fully represent exposure at the time of lung function measurement. If exposure misclassification is nondifferential (i.e., unrelated to lung function level), this would result in an attenuation of associations rather than generate false positives. However, we cannot rule out that for some exposures of interest, in particular for active smoking, misclassification might be differential (e.g., subjects with lower lung functions being more likely to quit smoking or subjects with higher lung functions being more likely to start smoking), in which case the direction of bias as a consequence of measurement error is less clear. The 2-year gap is not much of a concern for lifetime exposure trajectories, which are based on repeated measurements from birth to early adulthood. Lacking information on changes in exposure during these last 2 years is unlikely to affect exposure trajectory membership and consequently results. The consistency of findings for current exposure and life-course exposure trajectories argues against exposure misclassification being a major concern.

## Conclusions

In conclusion, our findings do not suggest adverse effects of the investigated indoor exposures on the maximum lung function level in early adulthood. A higher FVC was found in active smokers, which may be explained by bias arising from a selective uptake of smoking by individuals with higher pre-existing lung function.

## Conflict of interest statement

We declare the following potential conflicts of interest: G.H.K. reports grants from the Lung Foundation Netherlands, TEVA the Netherlands, VERTEX, European Union (Horizon grant), ZON-MW (VICI grant), and Growth Fund the Netherlands outside the submitted work. His institution received compensation for lectures and/or consultancy from AZ, Boehringer Ingelheim, Sanofi, and PURE-IMS. The other authors declare no competing interests.

## ACKNOWLEDGMENTS

*The authors would like to thank PIAMA participants who contributed to the study. We thank SURF* (www.surf.nl*) for the support in using the National Supercomputer Snellius.*

## Supplementary Material
