# Supplementary material for: Associations of current and life-course smoking and indoor environmental exposures with lung function level in early adulthood
Source: Environ Epidemiol. 2026 Aug 18;10(5):e523. doi: 10.1097/EE9.0000000000000523 (PMC13490050; doi:10.1097/EE9.0000000000000523)
Supplement: Supplementary file 1 [file ee9-10-e523-s001.pdf]

Supplementary Material

Associations of current and life-course smoking and indoor environmental exposures with lung function level in early adulthood

Diana M. Hendrickx[1], Gerard H. Koppelman[2,3], Judith M. Vonk[3,4], Jolanda M.A. Boer[5], Hans Jacob L. Koefoed[2,3], Marieke Oldenwening[1], Roel C.H. Vermeulen[1,6] and Ulrike Gehring[1,*]

[1] Institute for Risk Assessment Sciences (IRAS), Utrecht University, Utrecht, The Netherlands

[2] University of Groningen, University Medical Center Groningen, Beatrix Children's Hospital, Department of Paediatric Pulmonology and Paediatric Allergology, Groningen, The Netherlands

[3] University of Groningen, University Medical Center Groningen, Groningen Research Institute of Asthma and COPD (GRIAC), Groningen, The Netherlands

[4] University of Groningen, University Medical Center Groningen, Department of Epidemiology, Groningen, The Netherlands

[5] Centre for Prevention, Lifestyle and Health, National Institute for Public Health and the Environment (RIVM), Bilthoven, The Netherlands

[6] Julius Centre for Health Sciences and Primary Care, University Medical Centre Utrecht, Utrecht, The Netherlands

*Corresponding author: Address: Institute for Risk Assessment Sciences (IRAS), Utrecht University, Yalelaan 2, 3584 CM Utrecht, The Netherlands. Phone: +31 (0)6 39 58 69 68. Email: U.Gehring@uu.nl.

Table of Contents

Appendix A. Data collection instruments 5

Figure S1. Directed acyclic graph (DAG) illustrating potential covariates and assumptions about their relationships with active smoking, indoor exposures and lung function (DAG developed using DAGitty v3.1). 7

Figure S2. Flowchart of the number of participants in each analysis 8

Table S1. Number and percentage of surveys with missing data on active smoking and exposure to SHS, dampness or mould, pets and gas cooking (in the study population used to fit the exposure trajectories, N = 858). Total number of surveys: 5 on active smoking, 15 on SHS, 14 on dampness or mould, 15 on pets, 11 on gas cooking (+ for 3 ages derived from survey question on moving*) 9

Table S2. Comparison of characteristics between baseline population and the current study population. Bold = significant difference between baseline and current population. 10

Table S3. Prevalence of exposures at different time points in the population used for fitting trajectories (n/N (%)) 11

Table S4. Study characteristics by current active smoking status. Groups were compared using Chi-square tests. Bold = significant. 12

Table S5. Study characteristics by current SHS exposure status. Groups were compared using Chi-square tests. Bold = significant. 13

Table S6. Study characteristics by current dampness or mould exposure status. Groups were compared using Chi-square tests. Bold = significant. 14

Table S7. Study characteristics by current pet exposure status. Groups were compared using Chi-square tests. Bold = significant. 15

Table S8. Study characteristics by current gas cooking exposure status. Groups were compared using Chi-square tests. Bold = significant. 16

Table S9. Single exposure models - minimally adjusted associations of current active smoking and exposures to SHS, dampness or mould, furry pets (total), cats, dogs and gas cooking with pre- and post-bronchodilator FEV_1 and FVC at mean age 25 years expressed as estimated difference (mL) between exposed and non-exposed participants 17

Table S10. Multi-exposure model **-** additionally adjusted*associations of current active smoking and exposures to SHS, dampness or mould, furry pets (any) and gas cooking with pre- and post-bronchodilator FEV_1 and FVC at mean age 25 years expressed as estimated difference (mL) between exposed and non-exposed participants. 18

Table S11. Correlations between indoor exposures (phi-coefficient) of participants with post-bronchodilator FVC measurements. 19

Table S12. Variance inflation factors (VIF) of indoor exposures in the minimally and additionally adjusted models for post-bronchodilator FVC. 20

Table S13. Comparison of additionally adjusted* association of gas cooking exposure with post-bronchodilator FVC at mean age 25 between models (single-exposure model, two-exposure model gas cooking-active smoking, multi-exposure model (active smoking – SHS – dampness or mould – furry pets – gas cooking). Associations are expressed as estimated difference (mL) between exposed and non-exposed participants. 21

Table S14. Multi-exposure model - associations of current active smoking and exposures to SHS, dampness or mould, furry pets (total) and gas cooking with clinically low lung function, defined as having an pre-bronchodilator FEV_1 below the lower limit of normal (LLN). 22

Figure S3. Longitudinal trajectories of cat and dog exposure with trajectory frequencies. 23

Table S15. Study characteristics by active smoking trajectories. Groups were compared using Chi-square tests. Bold = significant. 24

Table S16. Study characteristics by SHS exposure trajectories. Groups were compared using Chi-square tests. Bold = significant. 25

Table S17. Study characteristics by dampness or mould exposure trajectories. Groups were compared using Chi-square tests. Bold = significant. 26

Table S18. Study characteristics by pet exposure trajectories. Groups were compared using Chi-square tests. Bold = significant. 27

Table S19. Study characteristics by gas cooking exposure trajectories. Groups were compared using Chi-square tests. Bold = significant. 28

Table S20. Minimally adjusted associations of longitudinal trajectories of active smoking, SHS, dampness or mould, furry pets (total), cats, dogs and gas cooking exposure with pre- and post-bronchodilator FEV1 and FVC at mean age 25 years. 29

Table S21. Associations of longitudinal trajectories of active smoking and exposures to SHS, dampness or mould, furry pets (total), cats, dogs and gas cooking with clinically low lung function, defined as having a pre-bronchodilator FEV_1 below the lower limit of normal (LLN). 30

Table S22. Single exposure models - additionally adjusted[*]associations of current active smoking and exposures to SHS, dampness or mould, furry pets (total) and gas cooking with pre-bronchodilator FEV_1 and FVC at mean age 25 years stratified by sex expressed as estimated difference (mL) between exposed and non-exposed participants. Bold = significant. 31

Table S23. Additionally adjusted[*] associations of longitudinal trajectories of active smoking and exposures to SHS, dampness or mould, furry pets (total) and gas cooking with pre-bronchodilator FEV1 and FVC at mean age 25 years stratified by sex. 32

Table S24. Single exposure models - additionally adjusted[*] associations of current active smoking and exposures to SHS, dampness or mould, furry pets (total) and gas cooking with pre-bronchodilator FEV_1 and FVC at mean age 25 years before and after excluding participants with asthma at age 23 expressed as estimated difference (mL) between exposed and non-exposed participants. 33

Table S25. Additionally adjusted[*] associations of longitudinal trajectories of active smoking and exposures to SHS, dampness or mould, furry pets (total) and gas cooking with pre-bronchodilator FEV_1 and FVC at mean age 25 years before and after excluding participants with asthma at age 23 expressed as estimated difference (mL) between exposed and non-exposed participants. 34

Table S26. Single exposure model **-** additionally adjusted[*] associations of current active smoking and exposures to SHS, dampness or mould, furry pets (total) and gas cooking with pre-bronchodilator FEV_1 and FVC at mean age 25 years before and after excluding participants with asthma during the first 4 years of live expressed as estimated difference (mL) between exposed and non-exposed participants. 35

Table S27. Additionally adjusted[*] associations of longitudinal trajectories of active smoking and exposures to SHS, dampness or mould, furry pets (total) and gas cooking with pre-bronchodilator FEV_1

and FVC at mean age 25 years before and after excluding participants with asthma during the first 4 years of live expressed as estimated difference (mL) between exposed and non-exposed participants. 36

Table S28. Single exposure models - additionally adjusted* associations of current active smoking and exposures to SHS, dampness or mould, furry pets (total) and gas cooking with pre-bronchodilator FEV_1 and FVC at mean age 25 years before and after excluding participants with parental asthma expressed as estimated difference (mL) between exposed and non-exposed participants. 37

Table S29. Additionally adjusted* associations of longitudinal trajectories of active smoking and exposures to SHS, dampness or mould, furry pets (total) and gas cooking with pre-bronchodilator FEV_1 and FVC at mean age 25 years before and after excluding participants with parental asthma expressed as estimated difference (mL) between exposed and non-exposed participants. 38

Table S30. Comparison of % predicted pre-bronchodilator FEV_1 and FVC (mean ± sd) between current, former and never smokers.. 39

Table S31. Comparison of % predicted pre-bronchodilator FEV1 and FVC (mean ± sd) at ages 8, 12 and 16 years between current, former and never smokers. 40

Appendix A. Data collection instruments

A.1. Questionnaires – subset of questions used to assess smoking and indoor exposures

A.1.1. Parental questionnaires

Question	Outcomes	prenatal	3 months	1 year	2 years	3 years	4 years	5 years	6 years	7 years	8 years	11 years	14 years	17 years
Do you smoke cigarettes?	1=yes; 2=no, in the past yes; 3=never	x												
When did you stop smoking?	1=more than a year ago; 2=less than a year ago	x												
How many months ago did you stop smoking?	1-12	x												
Does anyone smoke in your house?	1=yes; 2=yes, but less than once a week; 3=no seldom		x	x	x	x	x	x	x	x	x	x	x	x
Did you see any damp stains or mould spots on ceiling or walls of the living room in the past year?	1=yes 2=no		x			x	x	x	x	x	x	x	x	x
Did you see any damp stains or mold spots on ceiling or walls of the child's bedroom in the past year?	1=yes; 2=no		x			x	x	x	x	x	x	x	x	x
Have you seen any moisture stain or mould on the ceiling or walls of the living room since your child was born?	1=yes; 2=no			x	x									
Have you seen any moisture stain or mould on the ceiling or walls of the child's bedroom since your child was born?	1=yes;2=no			x	x									
Do you keep a dog in your home?	1=yes; 2=no	x	x	x	x	x	x	x	x	x	x	x	x	x
Do you keep a cat in your home?	1=yes; 2=no	x	x	x	x	x	x	x	x	x	x	x	x	x
Do you keep a rodent in your home?	1=yes; 2=no	x	x	x	x	x	x	x	x	x	x	x	x	x
Do you use natural gas for cooking?	1=yes; 2=no		x	x	x	x	x	x				x	x	x

A.1.2. Participant questionnaires

Question	Outcomes	11 years	14 years	17 years	20 years	23 years
Which of the following statement of smoking represents yourself?	1=smoke once a day<br>2=smoke not every day, but at least once a week<br>3=smoke not every week, but at least once a month<br>4=smoke less than once a month<br>5=try smoking now and then<br>6=stopped smoking after smoking at least once a week<br>7=stopped smoking, smoke always less than once a week<br>8=try smoking, but not anymore<br>9=never smoked	x	x			
Which statement about smoking suits you best?	1=every day<br>2=more than once a week<br>3=less than once a week<br>4=frequently, stopped now<br>5=tried, stopped<br>6=never			x	x	x
How often does someone smoke in your presence at home?	1=never<br>2=sometimes<br>3=about once a week<br>4=2-6 times a week<br>5=every day			x	x	x
Have you noticed damp or mouldy spots on walls or ceiling of living room in the past 12 months?	1=yes<br>2=no			x	x	x
Have you noticed damp or mouldy spots on walls or ceiling of bedroom in the past 12 months?	1=yes<br>2=no			x	x	x
In the home where you spend the largest part of your week, is there a dog?	1=yes<br>2=no			x	x	x
In the home where you spend the largest part of your week, is there a cat?	1=yes<br>2=no			x	x	x
In the home where you spend the largest part of your week, is there a rodent?	1=yes<br>2=no			x	x	x
Is gas used for cooking?	1=yes<br>2=no			x	x	x

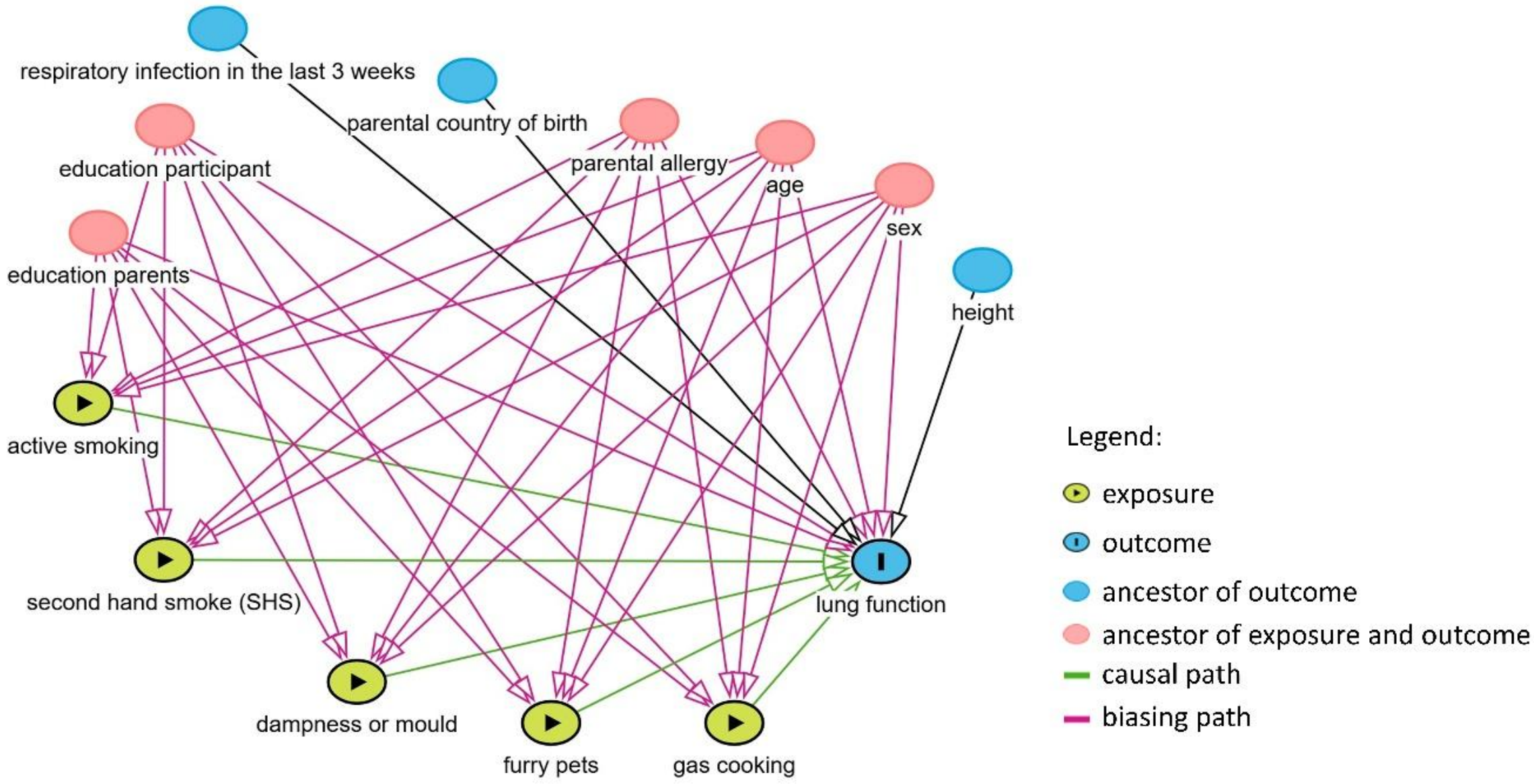

Figure S1. Directed acyclic graph (DAG) illustrating potential covariates and assumptions about their relationships with active smoking, indoor exposures and lung function (DAG developed using DAGitty v3.1).

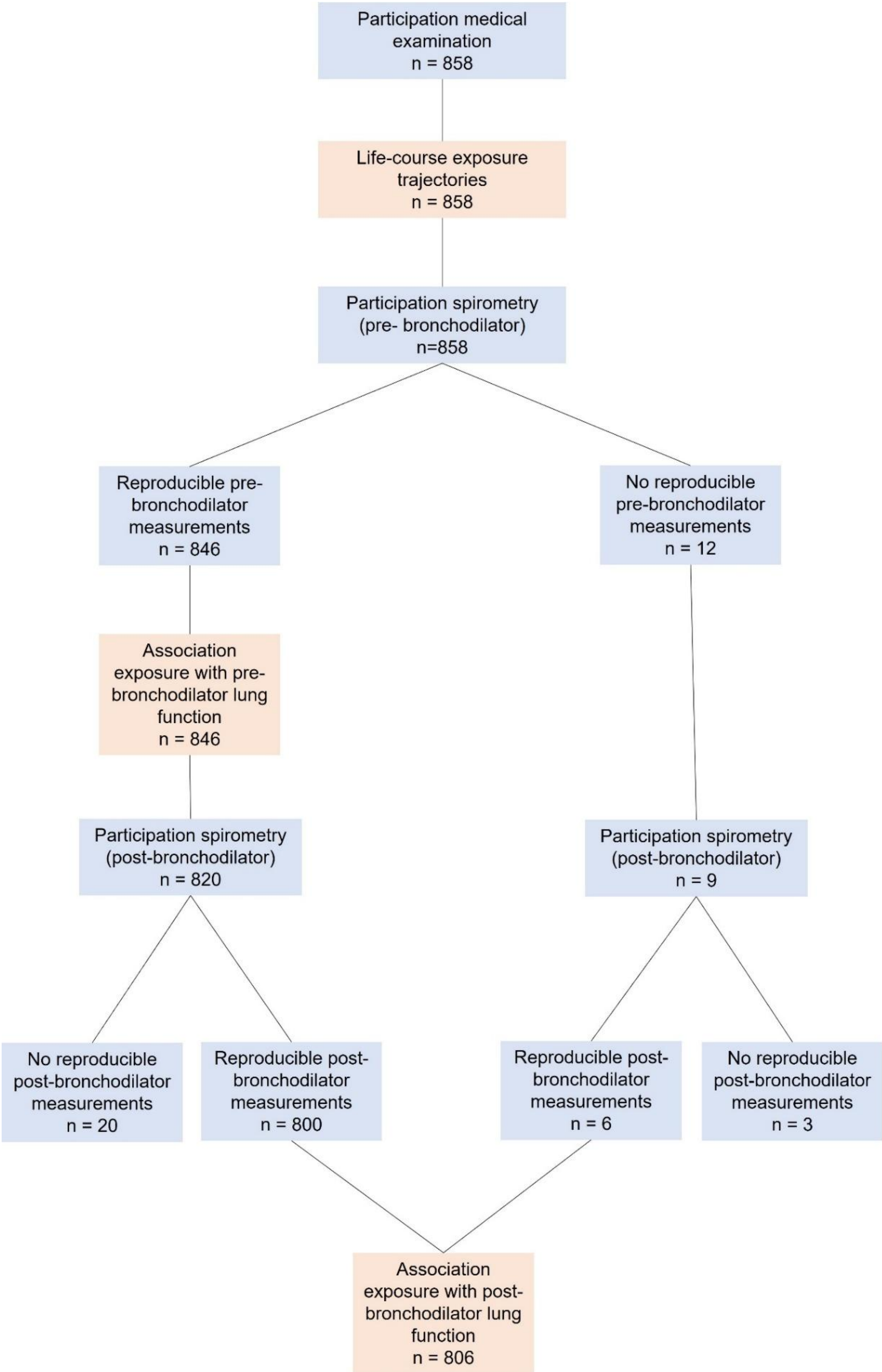

Figure S2. Flowchart of the number of participants in each analysis

Table S1. Number and percentage of surveys with missing data on active smoking and exposure to SHS, dampness or mould, pets and gas cooking (in the study population used to fit the exposure trajectories, N = 858). Total number of surveys: 5 on active smoking, 15 on SHS, 14 on dampness or mould, 15 on pets, 11 on gas cooking (+ for 3 ages derived from survey question on moving*).

Number of surveys with missing data	n (%)
**Active smoking**	
0	692 (80.7%)
1	110 (12.8%)
2	35 (4.1%)
3	18 (2.1%)
4	3 (0.3%)
**Second hand smoke (SHS)**	
0	629 (73.3%)
1	126 (14.7%)
2	52 (6.1%)
3	32 (3.7%)
4	8 (0.9%)
5	6 (0.7%)
6	3 (0.3%)
7	2 (0.2%)
**Dampness or mould**	
0	490 (57.1%)
1	208 (24.2%)
2	86 (10.0%)
3	36 (4.2%)
4	22 (2.6%)
5	6 (0.7%)
6	6 (0.7%)
7	3 (0.3%)
8	1 (0.1%)
**Furry pets**	
0	629 (73.3%)
1	115 (13.4%)
2	60 (7.0%)
3	33 (3.8%)
4	5 (0.6%)
5	11 (1.3%)
6	3 (0.3%)
8	1 (0.1%)
10	1 (0.1%)
**Gas cooking***	
0	640 (74.6%)
1	54 (6.3%)
2	50 (5.8%)
3	44 (5.1%)
4	17 (2.0%)
5	23 (2.7%)
6	16 (1.9%)
7	9 (1.0%)
8	1 (0.1%)
9	2 (0.2%)
10	1 (0.1%)
11	1 (0.1%)

***** after replacing NA by the value of the previous questionnaire if the participant still lives on the previous address or replacing NA by the value of the next questionnaire when the participant already lives on the next address.

Table S2. Comparison of characteristics between baseline population and the current study population. Bold = significant difference between baseline and current population.

	**baseline population (N = 3963)**	**Study population trajectories (N = 858)**		**Study population pre-bronchodilator (N = 846)**		**Study population post-bronchodilator (N = 806)**	
**covariates**	**n/N (%)**	**n/N (%)**	**p-value***	**n/N (%)**	**p-value***	**n/N (%)**	**p-value***
boy	2054/3963 (51.8%)	343/858 (40.0%)	**< 0.001**	338/846 (40.0%)	**< 0.001**	319/806 (39.6%)	**< 0.001**
parental country of birth (Netherlands)	3485/3700 (94.1%)	783/844 (92.7%)	0.129	770/832 (92.5%)	0.078	734/793 (92.6%)	0.086
parental allergy							
allergy mother	1237/3963 (31.2%)	269/858 (31.4%)	0.935	267/846 (31.6%)	0.838	258/806 (32.0%)	0.677
allergy father	1217/3957 (30.7%)	304/856 (35.5%)	**0.007**	300/844 (35.5%)	**0.007**	284/804 (35.3%)	**0.012**
parental education			**< 0.001**		**< 0.001**		**< 0.001**
low	502/3812 (13.1%)	58/848 (6.8%)		56/836 (6.7%)		55/797 (6.9%)	
intermediate	1402/3812 (36.7%)	247/848 (29.1%)		244/836 (29.2%)		231/797 (29.0%)	
high	1908/3812 (50.1%)	543/848 (64.0%)		536/836 (64.1%)		511/797 (64.1%)	

* comparison with the baseline population using a Fisher exact test

Table S3. Prevalence of exposures at different time points in the population used for fitting trajectories (n/N (%)).

	active smoking	SHS	dampness or mould	furry pets	cats	dogs	gas cooking*
**pregnancy**	-	97/850 (11.4%)	-	370/855 (43.3%)	252/856 (29.4%)	112/856 (13.1%)	-
**3 months**	-	191/858 (22.3%)	71/738 (9.6%)	364/858 (42.4%)	247/858 (28.8%)	114/858 (13.3%)	712/853 (83.5%)
**1 year**	-	179/857 (20.9%)	64/839 (7.6%)	353/853 (41.4%)	233/853 (27.3%)	111/854 (13.0%)	646/780 (82.8%)
**2 years**	-	178/851 (20.9%)	101/839 (12.0%)	353/846 (41.7%)	225/845 (26.6%)	100/847 (11.8%)	625/777 (80.4%)
**3 years**	-	169/853 (19.8%)	76/847 (9.0%)	355/845 (42.0%)	216/845 (25.6%)	93/846 (11.0%)	628/788 (79.7%)
**4 years**	-	145/840 (17.3%)	90/839 (10.7%)	384/837 (45.9%)	224/837 (26.8%)	101/838 (12.1%)	652/829 (78.6%)
**5 years**	-	138/842 (16.4%)	85/832 (10.2%)	383/833 (46.0%)	209/836 (25.0%)	109/836 (13.0%)	658/855 (77.0%)
**6 years**	-	129/842 (15.3%)	73/838 (8.7%)	382/844 (45.3%)	207/845 (24.5%)	121/846 (14.3%)	595/776 (76.7%)
**7 years**	-	110/836 (13.2%)	70/824 (8.5%)	391/834 (46.9%)	203/836 (24.3%)	124/837 (14.8%)	592/768 (77.1%)
**8 years**	-	93/823 (11.3%)	49/818 (6.0%)	401/820 (48.9%)	201/822 (24.5%)	129/822 (15.7%)	584/766 (76.2%)
**11 years**	0/788 (0.0%)	74/784 (9.4%)	63/782 (8.1%)	440/786 (56.0%)	215/787 (27.3%)	147/786 (18.7%)	599/808 (74.1%)
**14 years**	14/807 (1.7%)	58/766 (7.6%)	69/766 (9.0%)	427/766 (55.7%)	226/766 (29.5%)	179/766 (23.4%)	608/817 (74.4%)
**17 years**	65/789 (8.2%)	51/788 (6.5%)	68/720 (9.4%)	448/789 (56.8%)	242/789 (30.7%)	212/789 (26.9%)	618/831 (74.4%)
**20 years**	113/811 (13.9%)	47/811 (5.8%)	89/811 (11.0%)	296/811 (36.5%)	157/811 (19.4%)	149/811 (18.4%)	617/836 (73.8%)
**23 years**	102/849 (12.0%)	38/849 (4.5%)	118/848 (13.9%)	286/848 (33.7%)	177/848 (20.9%)	118/848 (13.9%)	553/853 (64.8%)

* after replacing NA by the value of the previous questionnaire if the participant still lives on the previous address or replacing NA by the value of the next questionnaire when the participant already lives on the next address.

Table S4. Study characteristics by current active smoking status. Groups were compared using Chi-square tests. Bold = significant.

**Characteristic**	**active smoking yes (%)**	**active smoking no (%)**	**P-value**
Sex			**0.046**
Males	49.0	38.2	
Females	51.0	61.8	
Parental country of birth			0.665
Netherlands	94.1	92.4	
Other	5.9	7.6	
Parental education			0.842
Low	6.1	7.0	
Middle	31.3	28.8	
High	62.6	64.2	
Parental allergy			0.140
Yes	49.0	57.3	
No	51.0	42.7	
Parental asthma			0.560
Yes	13.9	16.7	
No	86.1	83.3	

Table S5. Study characteristics by current SHS exposure status. Groups were compared using Chi-square tests. Bold = significant.

**Characteristic**	**SHS exposure yes (%)**	**SHS exposure no (%)**	**P-value**
Sex			**< 0.001**
Males	73.7	37.9	
Females	26.3	62.1	
Parental country of birth			0.629
Netherlands	89.2	92.7	
Other	10.8	7.3	
Parental education			0.447
Low	11.1	6.7	
Middle	33.3	28.9	
High	55.6	64.4	
Parental allergy			0.333
Yes	47.4	56.7	
No	52.6	43.3	
Parental asthma			0.440
Yes	10.5	16.6	
No	89.5	83.4	

Table S6. Study characteristics by current dampness or mould exposure status. Groups were compared using Chi-square tests. Bold = significant.

**Characteristic**	**dampness or mould exposure yes**	**dampness or mould exposure no**	**P-value**
Sex			**0.014**
Males	28.8	41.2	
Females	71.2	58.8	
Parental country of birth			
Netherlands			0.418
Other	94.8	92.2	
Parental education	5.2	7.8	
Low			0.485
Middle	4.2	7.2	
High	30.5	28.9	
Parental allergy	65.3	63.9	
Yes			0.861
No	55.1	56.4	
Parental asthma	44.9	43.6	
Yes			1.000
No	16.1	16.4	
	83.9	83.6	

Table S7. Study characteristics by current pet exposure status. Groups were compared using Chi-square tests. Bold = significant.

Characteristic	pet exposure yes	pet exposure no	P-value
Sex			0.605
Males	38.1	40.2	
Females	61.9	59.8	
Parental country of birth			0.344
Netherlands	94.0	91.9	
Other	6.0	8.1	
Parental education			**< 0.001**
Low	10.6	4.9	
Middle	40.8	23.2	
High	48.6	71.9	
Parental allergy			0.621
Yes	54.9	56.9	
No	45.1	43.1	
Parental asthma			**0.034**
Yes	20.4	14.4	
No	79.6	85.6	

Table S8. Study characteristics by current gas cooking exposure status. Groups were compared using Chi-square tests. Bold = significant.

Characteristic	gas cooking exposure yes	gas cooking exposure no	P-value
Sex			0.778
Males	39.4	40.7	
Females	60.6	59.3	
Parental country of birth			0.863
Netherlands	92.8	92.2	
Other	7.2	7.8	
Parental education			0.061
Low	5.3	9.4	
Middle	28.8	29.9	
High	65.9	60.7	
Parental allergy			0.136
Yes	58.2	52.7	
No	41.8	47.3	
Parental asthma			0.878
Yes	16.7	16.0	
No	83.3	84.0	

Table S9. Single exposure models - minimally adjusted associations of current active smoking and exposures to SHS, dampness or mould, furry pets (total), cats, dogs and gas cooking with pre- and post-bronchodilator FEV_1 and FVC at mean age 25 years expressed as estimated difference (mL) between exposed and non-exposed participants.

exposure (yes vs no)	**FEV_1: difference (mL) (95% CI)**		**FVC: difference (mL) (95% CI)**	
	**pre-bronchodilator (n = 846)**	**post-bronchodilator (n = 806)**	**pre-bronchodilator (n = 846)**	**post-bronchodilator (n = 806)**
**active smoking**	49 (-43 , 142)	**103 (14 , 193)**	**168 (63 , 274)**	**176 (67 , 284)**
**SHS**	31 (-116 , 177)	123 (-19 , 266)	150 (-19 , 319)	**243 (71 , 416)**
**dampness or mould**	12 (-75 , 99)	17 (-67 , 101)	-22 (-122 , 77)	-2 (-104 , 100)
**furry pets (total)**	-42 (-106 , 21)	-32 (-95 , 29)	-19 (-92 , 54)	-15 (-91 , 60)
**cats**	-61 (-134 , 12)	-51 (-123 , 20)	-12 (-98 , 73)	-6 (-94 , 81)
**dogs**	-14 (-102 , 74)	-3 (-88 , 82)	3 (-96 , 102)	-5 (-107 , 98)
**gas cooking**	-15 (-78 , 48)	-36 (-98 , 25)	-43 (-115 , 29)	-58 (-132 , 17)

Models were adjusted for sex, height, age and parental country of birth.
Bold = p-value < 0.05

Table S10. Multi-exposure model **-** additionally adjusted*associations of current active smoking and exposures to SHS, dampness or mould, furry pets (any) and gas cooking with pre- and post-bronchodilator FEV_1 and FVC at mean age 25 years expressed as estimated difference (mL) between exposed and non-exposed participants.

	**FEV_1: difference (mL) (95% CI)**		**FVC: difference (mL) (95% CI)**	
**Exposure (yes vs no)**	**pre-bronchodilator (n = 846)**	**post-bronchodilator (n = 806)**	**pre-bronchodilator (n = 846)**	**post-bronchodilator (n = 806)**
**Active smoking**	60 (-38, 157)	**106 (12, 200)**	**170 (59, 281)**	**167 (54, 281)**
**SHS**	13 (-140, 165)	93 (-56, 241)	100 (-76, 275)	**199 (19, 378)**
**Dampness or mould**	13 (-74,101)	18 (-67, 103)	-18 (-118, 82)	-2 (-104, 100)
**Furry pets (total)**	-41 (-107, 26)	-30 (-95, 34)	-22 (-97, 54)	-12 (-90, 65)
**Gas cooking**	-25 (-89, 40)	-54 (-117, 8)	-61 (-134, 12)	**-82 (-157, -7)**

*adjusted for sex, height, age, parental country of birth, parental allergy, cold or respiratory infection in the past three weeks, highest education of the parents and highest education of the participant.
Bold = p-value < 0.05

Table S11. Correlations between indoor exposures (phi-coefficient) of participants with post-bronchodilator FVC measurements.

	**active smoking**	**SHS**	**dampness or mould**	**furry pets**
**SHS**	0.27			
**dampness or mould**	0.00	0.03		
**furry pets**	0.04	0.01	-0.02	
**gas cooking**	0.10	0.07	0.07	-0.08

Table S12. Variance inflation factors (VIF) of indoor exposures in the minimally and additionally adjusted models for post-bronchodilator FVC.

exposure	VIF in minimally adjusted model	VIF in additionally adjusted model
**active smoking**	1.19328	1.22698
**SHS**	1.23052	1.24767
**dampness or mould**	1.03909	1.05001
**furry pets**	1.03313	1.19001
**gas cooking**	1.05342	1.07496

Table S13. Comparison of additionally adjusted* association of gas cooking exposure with post-bronchodilator FVC at mean age 25 between models (single-exposure model, two-exposure model gas cooking-active smoking, multi-exposure model (active smoking – SHS – dampness or mould – furry pets – gas cooking). Associations are expressed as estimated difference (mL) between exposed and non-exposed participants.

model	Post-bronchodilator FVC difference (mL) (95% CI) (n = 806)
Single-exposure	-62 (-137 , 13)
Two-exposure (gas cooking + active smoking)	**-77 (-152, -2)**
Multi-exposure	**-82 (-157, -7)**

*adjusted for sex, height, age, parental country of birth, parental allergy, cold or respiratory infection in the past three weeks, highest education of the parents and highest education of the participant.

Table S14. Multi-exposure model - associations of current active smoking and exposures to SHS, dampness or mould, furry pets (total) and gas cooking with clinically low lung function, defined as having an pre-bronchodilator FEV_1 below the lower limit of normal (LLN).

exposure (yes vs no)	odds ratio (95% CI) clinically low lung function	
	**Minimally adjusted* (n = 846)**	**Additionally adjusted# (n = 846)**
**active smoking**	1.31 (0.61, 2.78)	1.26 (0.58, 2.71)
**SHS**	1.84 (0.65, 5.16)	1.93 (0.68, 5.50)
**dampness or mould**	1.07 (0.51, 2.25)	1.06 (0.50, 2.24)
**furry pets (total)**	1.01 (0.57, 1.80)	1.09 (0.60, 1.98)
**gas cooking**	1.40 (0.77, 2.55)	1.41 (0.77, 2.57)

* adjustments for sex, height, age and ethnicity included in GLI equations

# adjusted for parental allergy, cold or respiratory infection in the past three weeks, highest education of the parents and highest education of the participant.

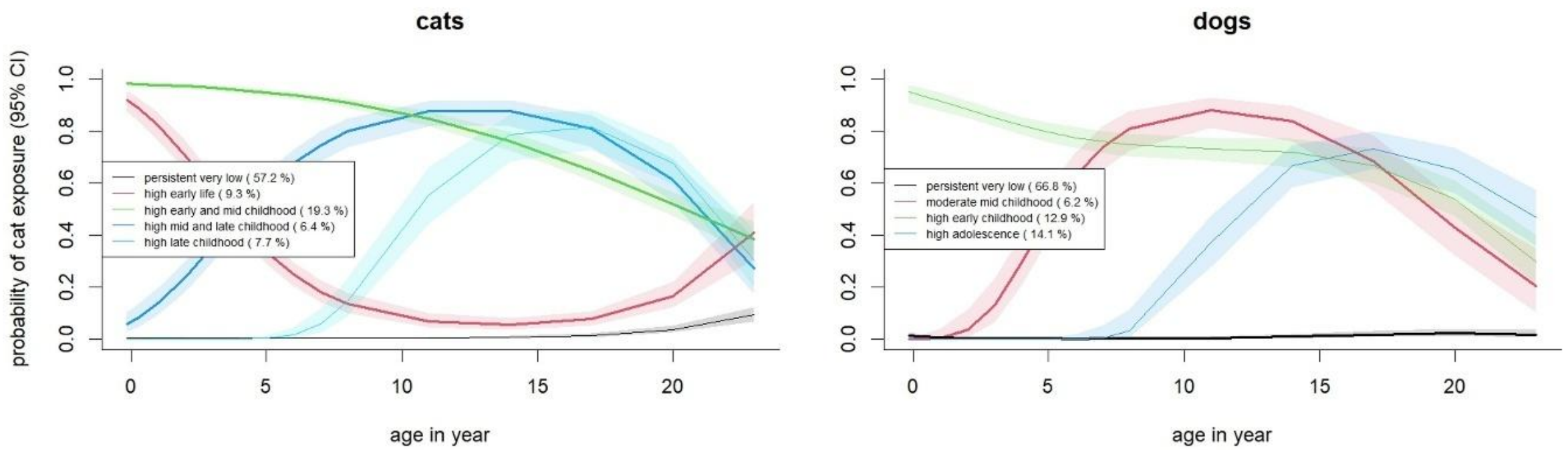

Figure S3. Longitudinal trajectories of cat and dog exposure with trajectory frequencies.

Table S15. Study characteristics by active smoking trajectories. Groups were compared using Chi-square tests. Bold = significant.

Characteristic	regular smoking (%)	occasional or no smoking (%)	P-value
Sex			**0.041**
Males	50.0	38.7	
Females	50.0	61.3	
Parental country of birth			0.877
Netherlands	91.8	92.8	
Other	8.2	7.2	
Parental education			0.792
Low	5.3	7.0	
Middle	30.9	28.9	
High	63.8	64.1	
Parental allergy			0.442
Yes	52.0	56.7	
No	48.0	43.3	
Parental asthma			0.705
Yes	14.6	16.7	
No	85.4	83.3	

Table S16. Study characteristics by SHS exposure trajectories. Groups were compared using Chi-square tests. Bold = significant.

**Characteristic**	**high childhood (%)**	**moderate early life (%)**	**very low (%)**	**P-value**
Sex				0.286
Males	44.4	44.3	38.4	
Females	55.6	55.7	61.6	
Parental country of birth				**0.019**
Netherlands	89.7	87.6	94.2	
Other	10.3	12.4	5.8	
Parental education				**< 0.001**
Low	17.8	13.4	3.7	
Middle	45.8	33.6	25.4	
High	36.4	52.9	70.9	
Parental allergy				**< 0.001**
Yes	37.0	57.4	59.2	
No	63.0	42.6	40.8	
Parental asthma				0.070
Yes	9.4	20.5	16.9	
No	90.6	79.5	83.1	

Table S17. Study characteristics by dampness or mould exposure trajectories. Groups were compared using Chi-square tests. Bold = significant.

Characteristic	moderate early childhood (%)	persistent low (%)	persistent very low (%)	P-value
Sex				0.116
Males	36.6	49.5	38.9	
Females	63.4	50.5	61.1	
Parental country of birth				0.800
Netherlands	95.1	91.9	92.6	
Other	4.9	8.1	7.4	
Parental education				0.389
Low	4.9	5.2	7.2	
Middle	19.5	25.8	30.1	
High	75.6	69.1	62.7	
Parental allergy				0.067
Yes	73.2	52.5	55.7	
No	26.8	47.5	44.3	
Parental asthma				0.087
Yes	26.8	20.2	15.3	
No	73.2	79.8	84.7	

Table S18. Study characteristics by pet exposure trajectories. Groups were compared using Chi-square tests. Bold = significant.

Characteristic	high late childhood (%)	high mid and late childhood (%)	high early and mid-childhood (%)	high early life (%)	moderate mid-childhood (%)	very low (%)	P-value
Sex							0.524
Males	40.7	45.2	40.5	41.4	44.3	35.1	
Females	59.3	54.8	59.5	58.6	55.7	64.9	
Parental country of birth							0.268
Netherlands	90.1	92.8	95.8	93.6	92.0	90.3	
Other	9.9	7.2	4.2	6.4	8.0	9.7	
Parental education	4.4	6.0	10.9	6.4	3.4	5.4	**< 0.001**
Low	22.0	34.9	37.4	37.6	14.8	23.0	
Middle	73.6	59.0	51.7	56.0	81.8	71.5	
High							
Parental allergy	57.1	58.3	48.3	59.5	55.7	61.6	0.088
Yes	42.9	41.7	51.7	40.5	44.3	38.4	
No							
Parental asthma	16.5	21.7	13.0	17.3	12.5	19.1	0.310
Yes	83.5	78.3	87.0	82.7	87.5	80.9	
No							

Table S19. Study characteristics by gas cooking exposure trajectories. Groups were compared using Chi-square tests. Bold = significant.

Characteristic	very high childhood and adolescence (%)	high adolescence (%)	high childhood moderate adulthood (%)	high early life and adulthood (%)	moderate adulthood (%)	P-value
Sex						0.091
Males	41.9	30.4	32.1	31.6	45.4	
Females	58.1	69.6	67.9	68.4	54.6	
Parental						
country of						0.841
birth	92.7	89.1	94.5	92.3	93.4	
Netherlands	7.3	10.9	5.5	7.7	6.6	
Other						
Parental						0.721
education	6.7	3.6	8.9	5.2	9.3	
Low	28.1	28.6	30.4	28.6	34.6	
Middle	65.2	67.9	60.7	66.2	56.1	
High						0.648
Parental allergy	56.4	64.3	50.0	55.7	54.6	
Yes	43.6	35.7	50.0	44.3	45.4	
No						**0.041**
Parental	15.9	30.9	10.7	15.2	15.7	
asthma	84.1	69.1	89.3	84.8	84.3	
Yes						
No						

Table S20. Minimally adjusted associations of longitudinal trajectories of active smoking, SHS, dampness or mould, furry pets (total), cats, dogs and gas cooking exposure with pre- and post-bronchodilator FEV1 and FVC at mean age 25 years.

**exposure**		**FEV_1: difference (mL) (95% CI)**		**FVC: difference (mL) (95% CI)**	
		**pre-bronchodilator (n = 846)**	**post-bronchodilator (n = 806)**	**pre-bronchodilator (n = 846)**	**post-bronchodilator (n = 806)**
**active smoking**	regular smoking vs occasional or no smoking	54 (-43 , 151)	94 (-1 , 189)	**163 (51 , 275)**	**165 (51 , 279)**
**SHS**	moderate early life vs very low	30 (-63 , 124)	26 (-66 , 117)	49 (-59 , 157)	41 (-69 , 152)
	high childhood vs very low	29 (-63 , 121)	28 (-64 , 119)	13 (-94 , 119)	9 (-102 , 120)
**dampness or mould**	persistent low vs persistent very low	-4 (-106 , 99)	-13 (-112 , 86)	4 (-111 , 119)	2 (-116 , 120)
	moderate early childhood vs persistent very low	44 (-130 , 217)	39 (-133 , 210)	172 (-26 , 371)	125 (-81 , 330)
**furry pets (total)**	moderate mid-childhood vs very low	20 (-113 , 153)	24 (-107 , 155)	0 (-154 , 154)	41 (-118 , 200)
	high early life vs very low	-2 (-110 , 107)	-10 (-116 , 96)	31 (-92 , 155)	15 (-112 , 142)
	high early and mid-childhood vs very low	-9 (-91 , 73)	-26 (-107 , 54)	-19 (-114 , 76)	-33 (-131 , 65)
	high mid and late childhood vs very low	-78 (-198 , 41)	-84 (-203 , 35)	-98 (-237 , 41)	-78 (-223 , 67)
	high late childhood vs very low	-17 (-141 , 107)	11 (-111 , 132)	20 (-125 , 166)	38 (-111 , 187)
**cats**	high early life vs persistent very low	5 (-104 , 115)	-20 (-127 , 88)	-2 (-128 , 125)	-20 (-150 , 110)
	high early and mid-childhood vs persistent very low	17 (-62 , 95)	5 (-71 , 82)	-25 (-115 , 66)	-29 (-122 , 65)
	high mid and late childhood vs persistent very low	-24 (-155 , 107)	-26 (-156 , 103)	-79 (-229 , 72)	-70 (-226 , 85)
	high late childhood vs persistent very low	24 (-96 , 143)	5 (-110 , 121)	1 (-137 , 139)	17 (-124 , 158)
**dogs**	moderate mid-childhood vs persistent very low	-48 (-181 , 85)	-25 (-156 , 106)	12 (-144 , 168)	27 (-132 , 187)
	high early childhood vs persistent very low	-59 (-151 , 31)	-67 (-157 , 22)	-27 (-133 , 79)	-47 (-157 , 62)
	high adolescence vs persistent very low	-23 (-117 , 71)	-19 (-113 , 74)	-37 (-148 , 73)	-34 (-149 , 81)
**gas cooking**	high early life and adulthood vs moderate adulthood	-88 (-226, 50)	-86 (-220 , 48)	-57 (-217 , 104)	-65 (-246 , 116)
	high childhood moderate adulthood vs moderate adulthood	-58 (-207 , 91)	-1 (-145 , 143)	-2 (-172 , 168)	31 (-143 , 204)
	high adolescence vs moderate adulthood	-106 (-263 , 52)	-78 (-238 , 83)	-58 (-247 , 131)	-29 (-230 , 171)
	very high childhood and adolescence vs moderate adulthood	-48 (-140 , 44)	-34 (-126 , 59)	-3 (-111 , 106)	2 (-117 , 120)

Models adjusted for sex, height, age and parental country of birth.
Bold = p-value < 0.05.

Table S21. Associations of longitudinal trajectories of active smoking and exposures to SHS, dampness or mould, furry pets (total), cats, dogs and gas cooking with clinically low lung function, defined as having a pre-bronchodilator FEV_1 below the lower limit of normal (LLN).

exposure	odds ratio (95% CI) clinically low lung function	
	**Minimally adjusted (n = 846)**	**Additionally adjusted[#] (n = 846)**
**active smoking**		
regular smoking vs occasional or no smoking	1.28 (0.57 , 2.89)	1.25 (0.55 , 2.82)
**SHS**		
moderate early life vs very low	0.53 (0.20 , 1.44)	0.55 (0.20 , 1.50)
high childhood vs very low	0.90 (0.40 , 2.04)	0.95 (0.40 , 2.24)
**dampness or mould**		
persistent low vs persistent very low	0.93 (0.37 , 2.31)	0.90 (0.36 , 2.23)
moderate early childhood vs persistent very low	1.29 (0.33 , 5.06)	1.24 (0.32 , 4.89)
**furry pets (total)**		
moderate mid-childhood vs very low	0.67 (0.20 , 2.20)	0.62 (0.19 , 2.07)
high early life vs very low	0.40 (0.12 , 1.26)	0.42 (0.13 , 1.34)
high early and mid-childhood vs very low	0.72 (0.35 , 1.46)	0.75 (0.36 , 1.55)
high mid and late childhood vs very low	1.29 (0.52 , 3.18)	1.36 (0.55 , 3.38)
high late childhood vs very low	0.58 (0.19 , 1.83)	0.57 (0.18 , 1.81)
**cats**		
high early life vs persistent very low	0.50 (0.15 , 1.72)	0.53 (0.15 , 1.82)
high early and mid-childhood vs persistent very low	1.06 (0.54 , 2.07)	1.10 (0.56 , 2.18)
high mid and late childhood vs persistent very low	1.81 (0.72 , 4.54)	1.92 (0.76 , 4.84)
high late childhood vs persistent very low	0.39 (0.08 , 1.86)	0.39 (0.08 , 1.83)
**dogs**		
moderate mid-childhood vs persistent very low	1.40 (0.53 , 3.68)	1.42 (0.54 , 3.77)
high early childhood vs persistent very low	0.45 (0.16 , 1.29)	0.48 (0.17 , 1.39)
high adolescence vs persistent very low	0.54 (0.21 , 1.41)	0.56 (0.21 , 1.46)
**gas cooking**		
high early life and adulthood vs moderate adulthood	2.23 (0.65 , 7.64)	2.19 (0.64 , 7.52)
high childhood moderate adulthood vs moderate adulthood	1.16 (0.25 , 5.34)	1.11 (0.24 , 5.10)
high adolescence vs moderate adulthood		
very high childhood and adolescence vs moderate	1.65 (0.35 , 7.74)	1.66 (0.35 , 7.90)
adulthood	1.71 (0.64 , 4.56)	1.64 (0.61 , 4.39)

[#] parental allergy, cold or respiratory infection in the past three weeks, highest education of the parents and highest education of the participant.

Table S22. Single exposure models - additionally adjusted* associations of current active smoking and exposures to SHS, dampness or mould, furry pets (total) and gas cooking with pre-bronchodilator FEV_1 and FVC at mean age 25 years stratified by sex expressed as estimated difference (mL) between exposed and non-exposed participants. Bold = significant.

**exposure (yes vs no)**	**pre-bronchodilator FEV_1: difference (mL) (95% CI)**			**pre-bronchodilator FVC: difference (mL) (95% CI)**		
	**Males (n = 338)**	**Females (n = 508)**	**Interaction p-value#**	**Males (n = 338)**	**Females (n = 508)**	**Interaction p-value#**
**active smoking**	80 (-83 , 243)	46 (-62 , 155)	0.479	**207 (27 , 387)**	**149 (19 , 279)**	0.415
**SHS**	24 (-182 , 230)	37 (-200 , 275)	0.987	188 (-42 , 417)	24 (-263 , 311)	0.431
**dampness or mould**	148 (-40 , 335)	-48 (-136 , 39)	**0.036**	125 (-82 , 116)	-85 (-191 , 21)	**0.049**
**furry pets (total)**	-58 (-188 , 73)	-11 (-81 , 59)	0.674	14 (-129 , 156)	-11 (-95 , 74)	0.821
**gas cooking**	-3 (-120 , 115)	-20 (-89 , 49)	0.678	-14 (-145 , 117)	-62 (-144 , 21)	0.402

* adjusted for height, age, parental country of birth, parental allergy, cold or respiratory infection in the past three weeks, highest education of the parents and highest education of the participant
# p-value obtained by including an interaction term exposure*sex in the model
Bold = p-value < 0.05.

Table S23. Additionally adjusted* associations of longitudinal trajectories of active smoking and exposures to SHS, dampness or mould, furry pets (total) and gas cooking with pre-bronchodilator FEV1 and FVC at mean age 25 years stratified by sex.

exposure	pre-bronchodilator FEV_1: difference (mL) (95% CI)			pre-bronchodilator FVC: difference (mL) (95% CI)		
	**Males (n = 338)**	**Females (n = 508)**	**Interaction p-value#**	**Males (n = 338)**	**Females (n = 508)**	**Interaction p-value#**
**active smoking**						
regular smoking vs occasional or no smoking	30 (-140 , 200)	96 (-18 , 209)	0.661	163 (-28 , 355)	**175 (39 , 311)**	0.946
**SHS**						
moderate early life vs very low	58 (-113 , 229)	41 (-66 , 148)	0.706	9 (-182 , 201)	106 (-22 , 235)	0.142
high childhood vs very low	118 (-53 , 290)	-29 (-138 , 81)	0.282	89 (-104 , 282)	-28 (-160 , 104)	0.675
**dampness or mould**						
persistent low vs persistent very low	-72 (-243 , 99)	57 (-61 , 175)	0.242	-40 (-228 , 149)	39 (-104 , 182)	0.585
moderate early childhood vs persistent very low	-117 (-467 , 232)	130 (-61 , 321)	0.219	43 (-375 , 461)	239 (-12 , 490)	0.494
**furry pets (total)**						
moderate mid-childhood vs very low	63 (-183 , 309)	-39 (-190 , 112)	0.462	-25 (-300 , 250)	5 (-177 , 187)	0.873
high early life vs very low	-42 (-241 , 157)	52 (-68 , 171)	0.442	-29 (-251 , 192)	106 (-37 , 250)	0.263
high early and mid-childhood vs very low	-47 (-213 , 119)	44 (-45 , 132)	0.327	-32 (-218 , 154)	36 (-71 , 143)	0.393
high mid and late childhood vs very low	-21 (-430 , 7)	55 (-83 , 194)	0.058	-227 (-474 , 20)	32 (-133 , 196)	0.090
high late childhood vs very low	34 (-197 , 266)	-27 (-161 , 108)	0.705	57 (-210 , 323)	23 (-138 , 185)	0.901
**gas cooking**						
high early life and adulthood vs moderate adulthood	-67 (-334 , 202)	-95 (-243 , 53)	0.785	-83 (-440 , 275)	-27 (-207 , 153)	0.828
high childhood moderate adulthood vs moderate adulthood	-250 (-547 , 47)	40 (-120 , 200)	0.087	-183 (-518 , 152)	91 (-111 , 293)	0.189
high adolescence vs moderate adulthood	-201 (-586 , 184)	-55 (-226 , 115)	0.478	-87 (-635 , 460)	-23 (-252 , 206)	0.813
very high childhood and adolescence vs moderate adulthood	-59 (-223 , 105)	-46 (-152 , 59)	0.983	-29 (-228 , 169)	13 (-117 , 142)	0.819

* adjusted for height, age, parental country of birth, parental allergy, cold or respiratory infection in the past three weeks, highest education of the parents and highest education of the participant
# p-value obtained by including an interaction term exposure*sex in the model
Bold = p-value < 0.05.

Table S24. Single exposure models - additionally adjusted* associations of current active smoking and exposures to SHS, dampness or mould, furry pets (total) and gas cooking with pre-bronchodilator FEV_1 and FVC at mean age 25 years before and after excluding participants with asthma at age 23 expressed as estimated difference (mL) between exposed and non-exposed participants.

exposure (yes vs no)	pre-bronchodilator FEV_1: difference (mL) (95% CI)		pre-bronchodilator FVC: difference (mL) (95% CI)	
	when including subjects with asthma (n = 846)	when excluding subjects with asthma (n = 772)	when including subjects with asthma (n = 846)	when excluding subjects with asthma (n = 772)
**active smoking**	56 (-37 , 150)	71 (-28, 169)	**175 (68 , 281)**	**176 (61, 291)**
**SHS**	33 (-114 , 181)	32 (-126, 191)	155 (-15 , 325)	124 (-63, 311)
**dampness or mould**	13 (-75 , 100)	22 (-68, 113)	-20 (-120 , 80)	-24 (-130, 82)
**furry pets (total)**	-39 (-105 , 28)	-55 (-124, 13)	-14 (-90 , 61)	-22 (-102, 58)
**gas cooking**	-17 (-80 , 47)	-18 (-84, 48)	-45 (-118 , 27)	-58 (-134, 19)

* adjusted for height, age, parental country of birth, parental allergy, cold or respiratory infection in the past three weeks, highest education of the parents and highest education of the participant

Bold = p-value < 0.05.

Table S25. Additionally adjusted* associations of longitudinal trajectories of active smoking and exposures to SHS, dampness or mould, furry pets (total) and gas cooking with pre-bronchodilator FEV_1 and FVC at mean age 25 years before and after excluding participants with asthma at age 23 expressed as estimated difference (mL) between exposed and non-exposed participants.

exposure	pre-bronchodilator FEV_1: difference (mL) (95% CI)		pre-bronchodilator FVC: difference (mL) (95% CI)	
	when including subjects with asthma* (n = 846)	when excluding subjects with asthma* (n = 772)	when including subjects with asthma* (n = 846)	when excluding subjects with asthma* (n = 772)
**active smoking**				
regular smoking vs occasional or no smoking	60 (-38 , 158)	65 (-37 , 168)	**168 (55 , 281)**	**174 (54 , 295)**
**SHS**				
moderate early life vs very low	38 (-58 , 133)	10 (-90, 111)	55 (-55, 165)	19 (-99 , 138)
high childhood vs very low	44 (-53, 141)	60 (-42 , 163)	33 (-79 , 145)	69 (-52 , 190)
**dampness or mould**				
persistent low vs persistent very low	-4 (-107 , 99)	-13 (-119, 93)	3 (-113 , 118)	0 (-122, 121)
moderate early childhood vs persistent very low	39 (-135 , 213)	92 (-87, 270)	162 (-38 , 362)	167 (-43, 377)
**furry pets (total)**				
moderate mid-childhood vs very low	19 (-115 , 153)	18 (-119, 155)	0 (-155 , 155)	-2 (-164, 160)
high early life vs very low	0 (-109 , 110)	28 (-86, 142)	37 (-87 , 162)	42 (-91, 175)
high early and mid-childhood vs very low	-5 (-89 , 78)	-3 (-89, 83)	-9 (-106 , 88)	-13 (-114, 88)
high mid and late childhood vs very low	-75 (-195 , 45)	-90 (-213, 33)	-93 (-232 , 47)	-112 (-257, 35)
high late childhood vs very low	-17 (-142 , 107)	7 (-123, 137)	21 (-125 , 168)	28 (-130, 186)
**gas cooking**				
high early life and adulthood vs moderate adulthood	-90 (-228 , 48)	-111 (-255, 33)	-58 (-219 , 103)	-62 (-230, 107)
high childhood moderate adulthood vs moderate adulthood	-54 (-203 , 96)	-61 (-211, 87)	0 (-171 , 170)	12 (-163, 188)
high adolescence vs moderate adulthood	-110 (-268 , 48)	-142 (-312, 28)	-64 (-252 , 125)	-86 (-291, 120)
very high childhood and adolescence vs moderate adulthood	-47 (-140 , 45)	-54 (-150, 42)	-5 (-114 , 104)	5 (-109, 118)

* adjusted for height, age, parental country of birth, parental allergy, cold or respiratory infection in the past three weeks, highest education of the parents and highest education of the participant
Bold = p-value < 0.05.

Table S26. Single exposure model **-** additionally adjusted* associations of current active smoking and exposures to SHS, dampness or mould, furry pets (total) and gas cooking with pre-bronchodilator FEV_1 and FVC at mean age 25 years before and after excluding participants with asthma during the first 4 years of live expressed as estimated difference (mL) between exposed and non-exposed participants.

**exposure (yes vs no)**	**pre-bronchodilator FEV_1: difference (mL) (95% CI)**		**pre-bronchodilator FVC: difference (mL) (95% CI)**	
	**when including subjects with asthma during the first 4 years of live (n = 846)**	**when excluding subjects with asthma during the first 4 years of live (n = 706)**	**when including subjects with asthma during the first 4 years of live (n = 846)**	**when excluding subjects with asthma during the first 4 years of live (n = 706)**
**active smoking**	56 (-37 , 150)	59 (-49 , 167)	**175 (68 , 281)**	**156 (34 , 279)**
**SHS**	33 (-114 , 181)	47 (-120 , 216)	155 (-15 , 325)	189 (-8 , 385)
**dampness or mould**	13 (-75 , 100)	6 (-86, 99)	-20 (-120 , 80)	-32 (-139, 76)
**furry pets (total)**	-39 (-105 , 28)	-21 (-92, 51)	-14 (-90 , 61)	-4 (-87, 80)
**gas cooking**	-17 (-80 , 47)	-14 (-83, 55)	-45 (-118 , 27)	-51 (-132, 29)

* adjusted for height, age, parental country of birth, parental allergy, cold or respiratory infection in the past three weeks, highest education of the parents and highest education of the participant
Bold = p-value < 0.05.

Table S27. Additionally adjusted* associations of longitudinal trajectories of active smoking and exposures to SHS, dampness or mould, furry pets (total) and gas cooking with pre-bronchodilator FEV_1 and FVC at mean age 25 years before and after excluding participants with asthma during the first 4 years of live expressed as estimated difference (mL) between exposed and non-exposed participants.

**exposure**	**pre-bronchodilator FEV_1: difference (mL) (95% CI)**		**pre-bronchodilator FVC: difference (mL) (95% CI)**	
	**when including subjects with asthma during the first 4 years of live (n = 846)**	**when excluding subjects with asthma during the first 4 years of live (n = 706)**	**when including subjects with asthma during the first 4 years of live (n = 846)**	**when excluding subjects with asthma during the first 4 years of live (n = 706)**
**active smoking**				
regular smoking vs occasional or no smoking	60 (-38 , 158)	59 (-50 , 168)	**168 (55 , 281)**	**151 (24 , 277)**
**SHS**				
moderate early life vs very low	38 (-58 , 133)	33 (-69 , 136)	55 (-55, 165)	34 (-85 , 154)
high childhood vs very low	44 (-53, 141)	54 (-53 , 161)	33 (-79 , 145)	31 (-94 , 157)
**dampness or mould**				
persistent low vs persistent very low	-4 (-107 , 99)	-29 (-139, 82)	3 (-113 , 118)	-34 (-160, 93)
moderate early childhood vs persistent very low	39 (-135 , 213)	27 (-173, 227)	162 (-38 , 362)	170 (-70, 411)
**furry pets (total)**				
moderate mid-childhood vs very low	19 (-115 , 153)	1 (-146, 149)	0 (-155 , 155)	-27 (-198, 145)
high early life vs very low	0 (-109 , 110)	22 (-98, 142)	37 (-87 , 162)	52 (-86, 189)
high early and mid-childhood vs very low	-5 (-89 , 78)	-10 (-102, 81)	-9 (-106 , 88)	-13 (-119, 94)
high mid and late childhood vs very low	-75 (-195 , 45)	-41 (-174, 91)	-93 (-232 , 47)	-77 (-232, 78)
high late childhood vs very low	-17 (-142 , 107)	-4 (-136, 128)	21 (-125 , 168)	38 (-118, 193)
**gas cooking**				
high early life and adulthood vs moderate adulthood	-90 (-228 , 48)	-121 (-271, 29)	-58 (-219 , 103)	-75 (-249, 99)
high childhood moderate adulthood vs moderate adulthood	-54 (-203 , 96)	-13 (-183, 156)	0 (-171 , 170)	-2 (-201, 197)
high adolescence vs moderate adulthood	-110 (-268 , 48)	-112 (-279, 54)	-64 (-252 , 125)	-95 (-291, 102)
very high childhood and adolescence vs moderate adulthood	-47 (-140 , 45)	-40 (-139, 59)	-5 (-114 , 104)	2 (-116, 120)

* adjusted for height, age, parental country of birth, parental allergy, cold or respiratory infection in the past three weeks, highest education of the parents and highest education of the participant
Bold = p-value < 0.05.

Table S28. Single exposure models - additionally adjusted* associations of current active smoking and exposures to SHS, dampness or mould, furry pets (total) and gas cooking with pre-bronchodilator FEV_1 and FVC at mean age 25 years before and after excluding participants with parental asthma expressed as estimated difference (mL) between exposed and non-exposed participants.

**exposure (yes vs no)**	**pre-bronchodilator FEV_1: difference (mL) (95% CI)**		**pre-bronchodilator FVC: difference (mL) (95% CI)**	
	**when including subjects with parental asthma (n = 846)**	**when excluding subjects with parental asthma (n = 707)**	**when including subjects with parental asthma (n = 846)**	**when excluding subjects with parental asthma (n = 707)**
**active smoking**	56 (-37 , 150)	84 (-19 , 187)	**175 (68 , 281)**	**197 (79 , 315)**
**SHS**	33 (-114 , 181)	53 (-107 , 212)	155 (-15 , 325)	**206 (22 , 390)**
**dampness or mould**	13 (-75 , 100)	26 (-72, 122)	-20 (-120 , 80)	-12 (-124, 100)
**furry pets (total)**	-39 (-105 , 28)	-43 (-119, 33)	-14 (-90 , 61)	-39 (-125, 47)
**gas cooking**	-17 (-80 , 47)	-17 (-88, 55)	-45 (-118 , 27)	-49 (-130, 33)

* adjusted for height, age, parental country of birth, parental allergy, cold or respiratory infection in the past three weeks, highest education of the parents and highest education of the participant
Bold = significant.

Table S29. Additionally adjusted* associations of longitudinal trajectories of active smoking and exposures to SHS, dampness or mould, furry pets (total) and gas cooking with pre-bronchodilator FEV_1 and FVC at mean age 25 years before and after excluding participants with parental asthma expressed as estimated difference (mL) between exposed and non-exposed participants.

exposure	pre-bronchodilator FEV_1: difference (mL) (95% CI)		pre-bronchodilator FVC: difference (mL) (95% CI)	
	**when including subjects with parental asthma (n = 846)**	**when excluding subjects with parental asthma (n = 707)**	**when including subjects with parental asthma (n = 846)**	**when excluding subjects with parental asthma (n = 707)**
**active smoking**				
regular smoking vs occasional or no smoking	60 (-38 , 158)	87 (-23 , 197)	**168 (55 , 281)**	**188 (63 , 314)**
**SHS**				
moderate early life vs very low	38 (-58 , 133)	76 (-32 , 185)	55 (-55, 165)	88 (-37 , 214)
high childhood vs very low	44 (-53, 141)	17 (-88 , 122)	33 (-79 , 145)	13 (-109 , 135)
**dampness or mould**				
persistent low vs persistent very low	-4 (-107 , 99)	14 (-101, 129)	3 (-113 , 118)	7 (-122, 137)
moderate early childhood vs persistent very low	39 (-135 , 213)	34 (-172, 239)	162 (-38 , 362)	156 (-83, 395)
**furry pets (total)**				
moderate mid-childhood vs very low	19 (-115 , 153)	50 (-101, 200)	0 (-155 , 155)	25 (-149, 199)
high early life vs very low	0 (-109 , 110)	-11 (-134, 112)	37 (-87 , 162)	25 (-116, 165)
high early and mid-childhood vs very low	-5 (-89 , 78)	-13 (-106, 81)	-9 (-106 , 88)	-37 (-145, 72)
high mid and late childhood vs very low	-75 (-195 , 45)	-78 (-217, 62)	-93 (-232 , 47)	-144 (-303, 15)
high late childhood vs very low	-17 (-142 , 107)	-19 (-158, 119)	21 (-125 , 168)	-2 (-168, 163)
**gas cooking**				
high early life and adulthood vs moderate adulthood	-90 (-228 , 48)	-91 (-245, 63)	-58 (-219 , 103)	-39 (-215, 138)
high childhood moderate adulthood vs moderate adulthood	-54 (-203 , 96)	-65 (-227, 97)	0 (-171 , 170)	4 (-184, 192)
high adolescence vs moderate adulthood	-110 (-268 , 48)	-108 (-297, 81)	-64 (-252 , 125)	-31 (-260, 198)
very high childhood and adolescence vs moderate adulthood	-47 (-140 , 45)	-55 (-158, 48)	-5 (-114 , 104)	16 (-104, 137)

* adjusted for height, age, parental country of birth, parental allergy, cold or respiratory infection in the past three weeks, highest education of the parents and highest education of the participant
Bold = significant.

Table S30. Comparison of % predicted pre-bronchodilator FEV_1 and FVC (mean ± sd) between current, former and never smokers.

smoker?	number (N = 858)	number with reproducible spirometry (N = 846)	% predicted FEV_1*	% predicted FVC#
current	102	99	97.02 +/- 12.37	101.70 +/- 10.77
former	78	77	97.34 +/- 9.80	101.25 +/- 9.67
never	669	661	95.67 +/- 9.97	98.21 +/- 10.02
unknown	9	9		

*% predicted FEV_1 = the percentage of the FEV_1 of a person compared to the expected FEV_1 for someone of the same age, sex, height and ethnicity (as determined by the GLI equations)

#% predicted FVC = the percentage of the FVC of a person compared to the expected FVC for someone of the same age, sex, height and ethnicity (as determined by the GLI equations)

Table S31. Comparison of % predicted pre-bronchodilator FEV1 and FVC (mean ± sd) at ages 8, 12 and 16 years between current, former and never smokers.

		Reproducible spirometry at age 8			Reproducible spirometry at age 12			Reproducible spirometry at age 16		
**Smoking status age 23**	**n**	**n**	**FEV_1 [% pred]***	**FVC [% pred]***	**n**	**FEV_1 [% pred]***	**FVC [% pred]***	**n**	**FEV_1 [% pred]***	**FVC [% pred]***
current	102	41	106.32 ± 11.16	105.50 ± 12.42	71	95.02 ± 10.62	98.21 ± 9.97	52	95.51 ± 11.71	97.95 ± 10.55
former	78	25	108.09 ± 7.91	104.13 ± 9.27	56	93.32 ± 9.65	96.33 ± 9.15	35	92.44 ± 9.57	96.24 ± 9.19
never	669	279	106.42 ± 10.77	104.36 ± 10.69	411	92.79 ± 9.91	95.28 ± 10.12	309	93.17 ± 10.64	94.88 ± 10.38

[$]of the 858 participants of the medical exam at mean age 25. For 9 participants, information on smoking status was missing.

* FEV_1 and FVC in % of what would be expected based on a person's age, sex, height and ethnicity , determined by the GLI equations[1]

References

1. Stanojevic S, Kaminsky DA, Miller MR, et al. ERS/ATS technical standard on interpretive strategies for routine lung function tests. *Eur Respir J*. 2022;60(1):2101499. doi:10.1183/13993003.01499-2021
